# Supplementary material for: Death of a parent during childhood and blood pressure in youth: a population-based cohort study of Swedish men
Source: BMJ Open. 2021 Apr 26;11(4):e043657. doi: 10.1136/bmjopen-2020-043657 (PMC8076918; doi:10.1136/bmjopen-2020-043657)
Supplement: Supplementary data [file bmjopen-2020-043657supp001.pdf]

Supplementary table 1. Association between the covariates and blood pressure (N=48624)

Covariates	Systolic blood pressure		Diastolic blood pressure		Hypertension
	LS Mean (95% CI)*	*P*-value*	LS Mean (95% CI)*	*P*-value*	OR (95% CI)*
Parental socioeconomic status					
Middle or high level non-manual employee	124.63 (124.41-124.85)	-	72.92 (72.75-73.10)	-	1.00
Low level non-manual employee	125.26 (124.94-125.59)	<0.01	72.93 (72.67-73.19)	0.99	1.09 (1.00-1.19)
Skilled worker	126.68 (126.46-126.91)	<0.01	72.92 (72.74-73.10)	0.98	1.25 (1.17-1.34)
Unskilled worker	126.48 (126.30-126.66)	<0.01	72.98 (72.83-73.12)	0.64	1.25 (1.17-1.33)
Farmer	127.59 (127.28-127.90)	<0.01	72.48 (72.24-72.73)	<0.01	1.51 (1.39-1.63)
Other	126.31 (125.62-127.00)	<0.01	73.34 (72.79-73.90)	0.16	1.28 (1.10-1.49)
Depression					
No	126.10 (126.00-126.21)	-	72.90 (72.81-72.98)	-	1.00
Yes	126.76 (125.91-127.61)	0.13	73.31 (72.63-73.98)	0.24	1.20 (1.01-1.42)
Anxiety					
No	126.10 (126.00-126.21)	-	72.90 (72.82-72.98)	-	1.00
Yes	128.90 (127.05-130.74)	<0.01	73.76 (72.30-75.23)	0.25	1.18 (0.81-1.71)

Cardiorespiratory fitness (stanine scores)					
1	124.62 (120.68-128.56)	0.38	74.41 (71.28-77.54)	0.29	1.43 (0.67-3.08)
2	124.57 (122.97-126.17)	0.03	72.43 (71.16-73.69)	0.64	0.89 (0.63-1.28)
3	125.48 (125.02-125.95)	<0.01	72.90 (72.53-73.27)	0.43	1.00 (0.89-1.12)
4	125.92 (125.65-126.19)	0.01	73.08 (72.87-73.30)	0.02	1.02 (0.94-1.10)
5	126.06 (125.84-126.27)	0.05	73.01 (72.84-73.18)	0.04	1.02 (0.95-1.09)
6	126.14 (125.90-126.38)	0.16	72.82 (72.63-73.01)	0.54	1.04 (0.97-1.12)
7	126.43 (126.13-126.73)	0.85	73.00 (72.76-73.24)	0.10	1.05 (0.97-1.15)
8	126.01 (125.68-126.35)	0.08	72.69 (72.42-72.96)	0.82	1.02 (0.93-1.12)
9	126.39 (126.13-126.65)	-	72.73 (72.53-72.94)	-	1.00
Number of personal friends					
0	124.97 (123.81-126.14)	0.07	72.61 (71.68-73.53)	0.56	0.78 (0.60-1.02)
1-3	126.25 (125.85-126.66)	0.37	72.75 (72.43-73.07)	0.44	1.00 (0.92-1.10)
3-5	126.26 (126.08-126.45)	0.09	72.89 (72.74-73.04)	0.94	1.02 (0.97-1.07)
>5	126.06 (125.93-126.20)	-	72.88 (72.77-72.99)	-	1.00
Confidential discussions with friends					

Never	126.45 (125.93-126.96)	<0.01	73.26 (72.85-73.66)	0.07	1.18 (1.06-1.32)
Sometime	126.36 (126.23-126.50)	<0.01	72.85 (72.74-72.95)	0.90	1.09 (1.04-1.14)
Quite often	125.68 (125.51-125.86)	-	72.86 (72.72-73.00)	-	1.00
Has a confidant					
No	126.05 (125.79-126.30)	0.46	72.99 (72.78-73.19)	0.24	0.97 (0.91-1.03)
Yes	126.15 (126.04-126.27)	-	72.85 (72.76-72.94)	-	1.00
Number of cigarettes smoked per day					
0	127.26 (127.10-127.42)	-	73.60 (73.47-73.73)	-	1.00
1-10	125.55 (125.37-125.74)	<0.01	72.46 (72.32-72.61)	<0.01	0.73 (0.69-0.77)
>10	125.05 (124.85-125.25)	<0.01	72.22 (72.06-72.38)	<0.01	0.66 (0.63-0.70)
Risky drinking behavior					
No	126.17 (126.06-126.28)	-	72.90 (72.81-72.98)	-	1.00
Yes	125.71 (125.42-126.00)	<0.01	72.96 (72.73-73.19)	0.63	0.91 (0.85-0.98)
Body mass index, kg/m^2†	-	-	-	-	1.13 (1.12-1.14)

LS Mean=least square mean; CI=confidence interval; OR=odds ratio.

*Unadjusted model.

†In the linear regression on body mass index and systolic blood pressure, the estimated parameter was 0.87 and *p*-value was <0.01; in the linear regression on body mass index and diastolic blood pressure, the estimated parameter was 0.40 and *p*-value was <0.01.

Supplementary table 2. Associations between death of a parent during childhood and least square means for systolic and diastolic blood pressure at conscription after excluding exposed study participants whose exposure status was defined using questionnaire data (N= 47968)

Type of exposure	Model 1*		Model 2†		Model 3‡	
	LS Mean (95% CI)	*P*-value	LS Mean (95% CI)	*P*-value	LS Mean (95% CI)	*P*-value
**Systolic blood pressure**						
Unexposed	126.09 (125.99-126.20)	-	126.12 (125.96-126.28)	-	127.81 (126.65-128.98)	-
Any loss	126.44 (126.01-126.87)	0.13	126.45 (126.02-126.88)	0.15	128.16 (126.93-129.39)	0.13
Cause of death of the parent						
Unnatural death	125.49 (124.58-126.40)	0.20	125.56 (124.65-126.47)	0.23	127.57 (126.10-129.03)	0.59
Cardiovascular death	126.94 (126.01-127.87)	0.08	126.99 (126.06-127.92)	0.07	128.40 (126.92-129.88)	0.22
Other natural cause	126.62 (126.05-127.19)	0.08	126.59 (126.02-127.16)	0.12	128.31 (127.02-129.59)	0.10
Gender of deceased parent						
Mother	126.45 (125.66-127.24)	0.38	126.35 (125.55-127.14)	0.57	128.13 (126.73-129.54)	0.43
Father	126.43 (125.92-126.94)	0.20	126.49 (125.98-127.00)	0.17	128.17 (126.91-129.43)	0.19
Child’s age at loss						
≤5 years	125.98 (124.90-127.07)	0.84	126.04 (124.95-127.13)	0.87	127.75 (126.17-129.33)	0.89

6-12 years	126.30 (125.57-127.03)	0.58	126.30 (125.57-127.03)	0.66	127.96 (126.59-129.33)	0.74
13-18 years	126.67 (126.06-127.28)	0.07	126.68 (126.06-127.30)	0.08	128.44 (127.13-129.75)	0.05
**Diastolic blood pressure**						
Unexposed	72.90 (72.82-72.99)	-	72.94 (72.81-73.07)	-	73.93 (72.99-74.87)	-
Any loss	72.98 (72.63-73.32)	0.70	72.95 (72.61-73.30)	0.95	73.93 (72.93-74.92)	0.99
Cause of death of the parent						
Unnatural death	72.16 (71.43-72.88)	0.05	72.10 (71.37-72.82)	0.02	73.20 (72.02-74.39)	0.06
Cardiovascular death	73.42 (72.68-74.16)	0.18	73.40 (72.66-74.14)	0.24	74.14 (72.94-75.33)	0.60
Other natural cause	73.13 (72.67-73.58)	0.34	73.12 (72.67-73.58)	0.46	74.14 (73.10-75.18)	0.40
Gender of deceased parent						
Mother	72.39 (71.76-73.02)	0.11	72.43 (71.79-73.06)	0.12	73.52 (72.39-74.66)	0.23
Father	73.22 (72.81-73.63)	0.14	73.17 (72.76-73.58)	0.27	74.09 (73.07-75.11)	0.43
Child's age at loss						
≤5 years	72.85 (71.99-73.72)	0.91	72.75 (71.87-73.62)	0.66	73.68 (72.40-74.95)	0.57
6-12 years	73.04 (72.46-73.62)	0.66	72.99 (72.40-73.57)	0.89	73.92 (72.81-75.02)	0.95
13-18 years	72.97 (72.49-73.45)	0.80	73.00 (72.50-73.49)	0.84	74.02 (72.97-75.08)	0.74

LS Mean=least square mean; CI=confidence interval.

*Model 1 was unadjusted.

†Model 2 was adjusted for parental socioeconomic status.

‡Model 3 was adjusted for parental socioeconomic status, depression, anxiety, body-mass index, cardiorespiratory fitness, number of friends, frequency of talking with friends confidentially, having a confidant, number of cigarettes smoked per day, and risky drinking behavior.

Supplementary table 3. Associations between death of a parent and blood pressure at conscription, stratified by parental socioeconomic status and childhood adversity (N=48624)

Potential effect modifiers	Systolic blood pressure		Diastolic blood pressure	
	LS Mean (95% CI)*	*P*-value†	LS Mean (95% CI)*	*P*-value†
Parental socioeconomic status				
Non-manual employee	126.75 (124.57-128.92)	-	73.61 (71.84-75.38)	-
Unskilled worker/Skilled worker	129.11 (127.53-130.70)	0.86	73.94 (72.66-75.22)	0.72
Famer/Other	129.61 (125.66-133.56)	0.64	76.09 (72.92-79.26)	0.01
Cumulative childhood adversity				
0	126.61 (122.78-130.44)	-	75.47 (72.38-78.56)	-
1	130.75 (128.58-132.92)	0.84	74.41 (72.66-76.16)	0.16
2	127.95 (125.56-130.35)	0.68	74.08 (72.16-76.00)	<0.01
≥3	127.37 (125.14-129.60)	0.96	73.00 (71.17-74.83)	0.13

LS Mean=least square mean; CI=confidence interval.

*Models were adjusted for parental socioeconomic status, depression, anxiety, body-mass index, cardiorespiratory fitness, number of friends, frequency of talking with friends confidentially, having a confidant, number of cigarettes smoked per day, risky drinking behavior and cumulative childhood experience.

†*P*-value for interaction between death of a parent and the potential effect modifier.
