# Supplementary material for: Death of a parent during childhood and blood pressure in youth: a population-based cohort study of Swedish men
Source: BMJ Open. 2021 Apr 26;11(4):e043657. doi: 10.1136/bmjopen-2020-043657 (PMC8076918; doi:10.1136/bmjopen-2020-043657)
Supplement: Author's manuscript [file bmjopen-2020-043657.draft_revisions.pdf]

BMJ Open is committed to open peer review. As part of this commitment we make the peer review history of every article we publish publicly available.

When an article is published we post the peer reviewers' comments and the authors' responses online. We also post the versions of the paper that were used during peer review. These are the versions that the peer review comments apply to.

The versions of the paper that follow are the versions that were submitted during the peer review process. They are not the versions of record or the final published versions. They should not be cited or distributed as the published version of this manuscript.

BMJ Open is an open access journal and the full, final, typeset and author-corrected version of record of the manuscript is available on our site with no access controls, subscription charges or pay-per-view fees (http://bmjopen.bmj.com).

If you have any questions on BMJ Open's open peer review process please email info.bmjopen@bmj.com

BMJ Open

Death of a parent during childhood and blood pressure in youth in men

Journal:	*BMJ Open*
Manuscript ID	bmjopen-2020-043657
Article Type:	Original research
Date Submitted by the Author:	10-Aug-2020
Complete List of Authors:	Chen, Hua; Karolinska Institutet, Department of Global Public Health<br>Hemmingsson, Tomas; Stockholm University, Department of Public Health Sciences; Karolinska Institutet, Institute of Environmental Medicine<br>Janszky, Imre ; Karolinska Institutet, Department of Global Public Health; Norwegian University of Science and Technology, Department of Public Health and General Practice, Faculty of Medicine<br>Rostila, Mikael ; Stockholm University, Department of Public Health Sciences; Centre for Health Equity Studies, Stockholm University and Karolinska Institutet<br>Forsell , Yvonne; Karolinska Institutet, Department of Global Public Health; Stockholm County Council, Centre for Epidemiology and Community Medicine<br>Meng, Linghui; Karolinska Institutet, Department of Global Public Health; Capital Institute of Pediatrics, Statistical Office<br>Liang, Yajun; Karolinska Institute, Department of Global Public Health<br>Laszlo, Krisztina; Karolinska Institutet, Department of Global Public Health
Keywords:	Hypertension < CARDIOLOGY, EPIDEMIOLOGY, MENTAL HEALTH

SCHOLARONE™
Manuscripts

I, the Submitting Author has the right to grant and does grant on behalf of all authors of the Work (as defined in the below author licence), an exclusive licence and/or a non-exclusive licence for contributions from authors who are: i) UK Crown employees; ii) where BMJ has agreed a CC-BY licence shall apply, and/or iii) in accordance with the terms applicable for US Federal Government officers or employees acting as part of their official duties; on a worldwide, perpetual, irrevocable, royalty-free basis to BMJ Publishing Group Ltd ("BMJ") its licensees and where the relevant Journal is co-owned by BMJ to the co-owners of the Journal, to publish the Work in this journal and any other BMJ products and to exploit all rights, as set out in our licence.

The Submitting Author accepts and understands that any supply made under these terms is made by BMJ to the Submitting Author unless you are acting as an employee on behalf of your employer or a postgraduate student of an affiliated institution which is paying any applicable article publishing charge ("APC") for Open Access articles. Where the Submitting Author wishes to make the Work available on an Open Access basis (and intends to pay the relevant APC), the terms of reuse of such Open Access shall be governed by a Creative Commons licence – details of these licences and which Creative Commons licence will apply to this Work are set out in our licence referred to above.

Other than as permitted in any relevant BMJ Author's Self Archiving Policies, I confirm this Work has not been accepted for publication elsewhere, is not being considered for publication elsewhere and does not duplicate material already published. I confirm all authors consent to publication of this Work and authorise the granting of this licence.

For peer review only

Death of a parent during childhood and blood pressure in youth in men

Hua Chen,[1]* Tomas Hemmingsson,[2,3] Imre Janszky,[1,4] Mikael Rostila,[2,5] Yvonne Forsell,[1,6] Linghui Meng,[1,7] Yajun Liang,[1] Krisztina D. László[1]

*Address correspondence to Hua Chen, Department of Global Public Health, Karolinska Institutet, Tomtebodavägen 18A, Stockholm, 171 77, Sweden. E-mail: hua.chen@ki.se

For peer review only

ABSTRACT

Objective Compelling evidence suggests that childhood adversities are associated with an increased risk of hypertension in middle- and old age. The link between childhood adversities and blood pressure in youth is less clear. In this cohort study, we examined the association between death of a parent during childhood and blood pressure in early adulthood in men.

Setting Sweden.

Participants We studied 48,624 men born in 1949-51 who participated in the compulsory military conscription in 1969/70 in Sweden. Information on death of a parent during childhood was obtained from population-based registers. Information on covariates was obtained from the questionnaire and the clinical examination completed at conscription and from population-based registers.

Outcome measures Blood pressure was measured at conscription according to standard procedures.

Results The multivariable least square means of systolic and diastolic blood pressure did not differ between bereaved [128.25 (127.04-129.46) and 73.86 (72.89-74.84) mmHg] and non-bereaved study participants [128.02 (126.86-129.18) and 73.99 (73.06-74.93) mmHg]. Results were similar when considering the cause of the parent's death, the gender of the deceased parent, or the child's age at loss. Loss of a parent in childhood tended to be associated with an increased hypertension risk [odds ratio and 95% confidence interval: 1.10 (1.00-1.20)]; the association was present only in case of natural deaths.

Conclusion We found no strong support for the hypothesis that stress following the loss of a parent during childhood is associated with blood pressure or hypertension in youth in men.

For peer review only - http://bmjopen.bmj.com/site/about/guidelines.xhtml

Strengths and limitations of this study

- This is the first study to investigate prospectively the association between parental death in childhood and blood pressure measured in early adulthood in a large national cohort; the prospective design limited recall bias, while the large national sample allowed to reduce selection bias and to conduct sub-analyses to test hypotheses that may be supportive of a causal effect, if any.
- Since only one or two blood pressure measurements were taken, there was no possibility to consider intra-individual variations in blood pressure or to reduce the impact of white-coat hypertension.
- Information on some potential confounders was not available.
- It is not clear to what extent our findings may be generalizable to women, to current Swedish young adults or to populations with different risk factor profile, support for bereaved children and healthcare.

For peer review only

INTRODUCTION

Death of a parent is one of the most traumatic and stressful events that a child can experience.[1] In the Nordic countries around 4% of children experience parental death before the age of 18 years,[2 3] while in the developing countries the prevalence is higher.[4 5] Children exposed to parental death have an increased risk of experiencing poor social support,[6] heightened stress reactivity,[7] poor school performance,[8] emotional and behavioral problems,[6] psychiatric disorders,[9 10] substance use,[11 12] obesity[13] and/or metabolic syndrome;[14] these in turn may induce stress-related physiological and lifestyle changes that impact cardiovascular function and increase blood pressure (BP) already in young age.

To our knowledge, only five studies investigated the association between death of a parent and BP and the findings have been inconsistent.[4 7 15-17] Luecken[7] and Anderson[16] reported that study participants who experienced the death of a parent during childhood had higher systolic BP (SBP) and diastolic BP (DBP) than their unexposed counterparts. In contrast, Schooling[4] and Stein[17] found no association between death of a parent and BP or hypertension. In a study conducted later, Luecken et al. found that university students who lost their parent had lower 24-hour ambulatory BP compared to non-bereaved students.[15] None of these studies considered the importance of the cause of the parent's death, which limited the possibilities to address whether an eventual association was due to confounding by genetic or environmental cardiovascular factors shared by family members or also due to stress. Furthermore, except for the studies conducted by Luecken et al., which assessed BP in childhood or in young adulthood (age 18-29 years), all other studies were conducted in middle-aged individuals and assessed the exposure retrospectively;[7 15] thus they could not eliminate selection bias, i.e., that severe hypertensive patients may die before middle-age.

High BP in youth tracks into adulthood and is an important predictor of later hypertension and cardiovascular diseases (CVD);[18 19] it is associated with increased risks of abnormal left

For peer review only - http://bmjopen.bmj.com/site/about/guidelines.xhtml

ventricular mass,[20] metabolic syndrome,[21] impaired cognitive ability,[22] renal damage[23] and cardiovascular and total mortality.[24 25] The prevalence of elevated BP among children and young adults has been increasing.[26 27] Though adverse childhood experiences (most often defined in terms of maltreatment and family dysfunction), are associated with an increased risk of hypertension in middle- and old age,[28-30] knowledge about the association between childhood adversities and high BP in youth is more limited.[31-34]

In this study, we investigated the association between death of a parent during childhood − one of the most severe childhood adversities − and BP at the age of 18-20 years in a cohort of Swedish men participating in mandatory conscription. We also analyzed whether this association differs by the parent's cause of death, the gender of the deceased parent and the child's age at loss.

METHODS

Study population and design

This study was based on a cohort consisting of 49,321 men born in 1949-51 and who were conscripted for military service in Sweden in 1969 or 1970. The cohort includes 97-98% of all Swedish men of conscription age (≈18-20 years); the 2-3% that were exempted from participation had severe congenital disorders or intellectual disabilities.[35] At conscription, participants completed an extensive questionnaire and participated in a clinical examination performed by a team of physicians, psychiatrists and psychologists. The data collected at conscription were linked to several socioeconomic and health-related nationwide registers through the unique personal identification number. We excluded participants with no information on exposure (n=314), missing BP (n=369) or higher DBP than SBP (n=14), resulting in 48,624 men being included in our analysis.

For peer review only - http://bmjopen.bmj.com/site/about/guidelines.xhtml

Measures

Exposure. Conscripts were linked to their parents using the Multigeneration Register. We obtained information on the date and the cause of the parent's death after 1952 from the Cause of Death Register; causes of death were recorded according to the International Classification of Diseases (ICD) (6th revision during 1952-1957, 7th revision during 1958-1968, and 8th revision during 1969-1986). We classified the cause of the parent's death as death due to unnatural causes (ICD-6: 795 and 800-999; ICD-7: 795 and 800-999; ICD-8: 79599, 796 and 807-999), death due to non-rheumatic CVD (ICD-6: 400-468; ICD-7: 400-468; ICD-8: 400-458) and death due to other natural causes (the rest of the ICD codes). For the conscripts whose parents did not have a record in the Cause of Death Register, we used two items from the questionnaire completed at conscription to determine their exposure status before 1952: "Are both of your parents alive" (yes/no), and "Whom have you mostly lived with" (with both parents/with mother/with father/with somebody else). If study participants responded "no" to the first question, the gender of the deceased parent was determined using the second question: (1) having lived with the mother was coded as death of the father, (2) having lived with the father was coded as death of the mother, and (3) having lived with both parents or somebody else was coded as having no information on the gender of the deceased parent.

Outcome. Blood pressure was measured at conscription according to a written protocol; the measurement was performed in a supine position, after a 5-10 minutes rest, with an appropriately sized cuff at the heart level.[35-37] Thus the measurement of BP in this study reflects the standard practice in primary care settings.[37] The measured SBP and the DBP values were rounded to the nearest even number, or to the nearest 5 or 10 mmHg. If the SBP was higher than 145 mmHg or if the DBP was lower than 50 mmHg or higher than 85 mmHg, another measurement was conducted on the next day and the result of the second measurement was recorded; otherwise only one BP measurement was taken. Given that there is a linear

For peer review only - http://bmjopen.bmj.com/site/about/guidelines.xhtml

association between BP in young age and the later risk of CVD,[38] that even BP values below 140/90 mmHg have a predictive value for the development of later CVD, and since both SBP and DBP are associated with later CVD risk,[39 40] we considered the continuous SBP and DBP measures as the main outcome. In sensitivity analyses we also performed analyses with hypertension, defined as SBP ≥140 mmHg and/or DBP ≥90 mmHg.[41]

Covariates (confounders, mediators and/or effect modifiers). The occupation of the head of the household (in most cases the father) from the National Population and Housing Censuses of 1960 was used as a measure of parental socioeconomic status (SES). Occupation was classified as: (1) non-manual at middle or high level, (2) non-manual employee at low level, (3) skilled worker, (4) unskilled worker, (5) farmer, and (6) other.[42] Men who during the interview with the psychologist reported or were suspected to have a psychiatric disorder were referred to a psychiatrist for further evaluation; eventual psychiatric diagnoses were recorded according to ICD-8. We retrieved information on depression using ICD-8 codes 296 and 300.4 and information on anxiety using the ICD-8 code 300.0, respectively. We calculated body mass index based on height and weight assessed at the clinical examination. Cardiorespiratory fitness was assessed using a cycle ergometer and the resulting maximal work capacity divided by body weight was transformed to stanine scores (1-9).[36] The number of personal friends (0, 1-3, 3-5 or >5), the frequency of confidentially discussions with friends (never, sometimes or quite often), having a confidant to talk about personal problems (no one, or parent/siblings/teachers or supervisor or manager/friends/others), number of cigarettes per day (>10, 1-10, or 0), risky drinking behavior (defined as reporting any of the following: drinking more than 250 g per week, having taken alcohol as an 'eye-opener' during a hangover, having been arrested for drunkenness, or having often been intoxicated)[43] and father's drinking habit (often, or sometimes/occasionally/never) were assessed by questionnaires. We calculated a cumulative index of childhood adversities by summing the number of study participants' adverse childhood

For peer review only - http://bmjopen.bmj.com/site/about/guidelines.xhtml

experiences reported by questionnaire from the following: poor financial situation of the family (very good/good/medium versus bad/very bad), paternal alcohol use (often versus never/occasionally/sometimes), parents' divorce (yes versus no), severe illness of parent (father/mother/both parents versus none), family member taking medicines for mental disorders (mother/father/both parents/others versus no one), experiencing physical punishment (often/sometimes/once in a while versus never), strict upbringing (very strict/quite strict versus medium/quite mild/very mild), and having multiple residences during childhood (more than three residences versus one/two/three residences).

Statistical analyses

We compared characteristics of men exposed and not exposed to death of a parent during childhood by chi-square tests in case of categorical variables and by Wilcoxon tests in case of continuous variables with a skewed distribution. We used general linear regression models to investigate the association between death of a parent during childhood and the continuous SBP and DBP measures. For each exposure category, we also estimated least square means of SBP and DBP and their 95% confidence intervals (CI). We performed analyses with any death of a parent during childhood and with exposure classified based on the cause of the parent's death (due to CVD, other natural death or unnatural death), the gender of the deceased parent (mother or father), and the age of the child when the parent died (0-5, 6-12, 13-18 years). We ran three models. Model 1 was unadjusted. Model 2 was adjusted for childhood parental SES, a factor that is likely to be confounder of the investigated association. Model 3 was further adjusted for depression, anxiety, body mass index, cardiorespiratory fitness, number of friends, frequency of talking with friends confidentially, having a confidant, number of cigarettes smoked per day, and risky drinking behavior. We chose to run model 3 as separate models since we could not determine whether the variables in model 3 are confounders or mediators of the association

For peer review only - http://bmjopen.bmj.com/site/about/guidelines.xhtml

under study; though their assessment after the exposure period may favor regarding them as mediators, several of these characteristics could have been present before the loss of a parent and may thus be confounders. To study whether father's drinking behavior confounded the association between death of a father and BP, we re-ran the analyses regarding the association between death of a father and BP after adjusting for father's drinking behavior in addition to factors in model 3. To assure that the method of exposure assessment did not influence our results, we ran sensitivity analyses in which we excluded the conscripts whose parent's death was identified only by questionnaire. We investigated effect modification by parental SES (classified as (1) non-manual employee versus (2) unskilled workers or skilled workers versus (3) famer or whether occupations) and by our cumulative index of adverse life experiences (0, 1, 2 or ⩾3) by conducting stratified analyses and formal tests of interaction. In further sensitivity analyses we run logistic regression models to investigate the association between death of a parent (any loss and exposure classified by the parent's cause of death, the deceased parent's gender and the child's age at loss) and hypertension. We used listwise deletion if there is missing information of covariates.

We used SAS 9.4 for Windows for all statistical analyses.

RESULTS

Of the 48624 men included in our study, 3504 (7.21%) experienced death of a parent before the age of 18. Compared to their unexposed counterparts, bereaved study participants were more likely to have lower parental SES during childhood, worse cardiorespiratory fitness, less frequent confidential discussions with friends, no confidant, higher number of adverse childhood experiences, risky drinking behaviors, and fathers who drank alcohol frequently; furthermore, the bereaved study participants were more likely to be diagnosed with depression and to be smokers (Table 1).

For peer review only - http://bmjopen.bmj.com/site/about/guidelines.xhtml

The unadjusted and the adjusted least square means of SBP and DBP were generally similar between the exposed and the unexposed groups, both when investigating any loss of a parent and when the exposure was classified according to the deceased parent's cause of death and gender, or the child's age at loss (Table 2). Men whose mother died due to unnatural causes had a slightly lower DBP than their unexposed counterparts in the unadjusted and the SES-adjusted, but not in the fully adjusted models (Table 3). The results did not change substantially after excluding conscripts whose parent's death was defined through questionnaire-based information (Supplementary table 1). We found no strong evidence that parental SES or cumulative childhood adversity modified the association between death of a parent and SBP or DBP (Supplementary table 2). Adjusting for father's drinking behavior in addition to factors in model 3 did not substantially change the association between death of a father and SBP or DBP (data not shown).

Death of a parent tended to be associated with a slightly increased odds of hypertension; the corresponding multivariate-adjusted odds ratio (OR) (95% CI) was 1.10 (1.00-1.20). Losing a parent due to natural causes other than CVD was associated with hypertension (multivariate-adjusted OR (95% CI): 1.20 (1.06-1.35)). Losing a parent due to CVD also tended to be associated with hypertension (adjusted OR (95% CI): 1.15 (0.95-1.40)). There was no association between death of a parent due to unnatural causes and the risk of hypertension. The point estimates corresponding to the association between death of a parent and hypertension did not differ substantially according to the parent's gender. Risks were slightly higher in case the child was 6-12 or 13-18 years at the time of the loss compared to when the loss occurred at earlier ages; these associations were confined only to losses due to natural causes (Table 4).

DISCUSSION

For peer review only - http://bmjopen.bmj.com/site/about/guidelines.xhtml

We found no strong evidence for an association between the death of a parent during childhood and SBP or DBP in early adulthood. The associations did not differ by the parent's cause of death, the gender of the deceased parent or the child's age at loss. Losing a parent was associated with a slightly increased risk of hypertension; the association was present only in case of losses due to natural causes.

Earlier studies investigating the association between death of a parent during childhood and elevated BP or hypertension have yielded mixed results. Two studies observed higher SBP and DBP among study participants exposed to the death of a parent during childhood than among their unexposed counterparts.[7 16] Two other studies found no relation between death of a parent and BP or hypertension,[4 17] whereas one study observed that students who experienced the death of a parent had lower 24-hour ambulatory BP than non-bereaved students.[15] The reasons for these inconsistent findings are not clear but could be due to differences in study design, sample size, historical birth cohort, the age and the method of BP assessment and considerations about confounding by design or in multivariate models. Findings concerning the association between other types of childhood adversities – often defined as a cumulative index of events related to abuse, neglect or family dysfunction – and hypertension in middle- or old age are more consistent,[28-30] possibly due to the inclusion of a larger number of adverse life events and biases associated with the retrospective assessment of exposure. Our study extends knowledge in this field by (1) focusing on BP assessed at a young age, (2) using prospectively recorded information on parent's death from a high-quality nationwide register, rather than relying on retrospectively collected, questionnaire-based information on exposure, and (3) analyzing a very large sample that allowed us to conduct sub-analyses to test hypotheses that may be supportive of a causal effect, if any.

An important challenge in studies regarding the association between bereavement and high BP and other CVDs is related to the separation of the stress-related effect from confounding by

For peer review only - http://bmjopen.bmj.com/site/about/guidelines.xhtml

genetic and environmental cardiovascular risk factors shared by family members. The effect of exposure to death of a relative due to natural causes, in particular those due to CVD, on the risk of high BP is likely to be confounded by genetic and environmental cardiovascular factors shared by family members; the importance of familial confounding by cardiovascular risk factors is likely to be substantially more modest in case of unnatural deaths.[2 44] In contrast, unnatural deaths have been suggested to be more strongly associated with stress and an increased risk of complicated grief than deaths due to natural causes.[6 45 46] Furthermore, given that in most cultural contexts mothers have stronger emotional bonds with their children, are more involved in their children's upbringing,[47] have a stronger impact on their children's health behavior and are likely to provide more emotional support for coping with grief than fathers,[48] we expected that maternal death may have a more negative effect on youth BP than paternal death. Finally, we also hypothesized that the first few years of life and adolescence could be two sensitive periods with respect to the health consequences of losing a parent. Losing a parent in the first few years of life may be particularly detrimental[49] as warm relationships with caregivers are critical for the development of the brain architecture and the programming of stress reactivity,[7] while loss of a parent in adolescence, another period with an increased stress-sensitivity, may result in taking up adverse health behaviors that increase the risk of CVD.[50] Nevertheless, we found no association between death of a parent during childhood and our continuous BP measures neither in case of overall exposure, nor when exposure was categorized according to the parents' cause of death, parent's gender or the child's age at loss.

The finding that a modestly increased risk of hypertension was observed only in case of parental deaths due to natural causes but not in case of parental deaths due to unnatural causes − which are often associated with more severe stress[6 45 46] – suggests that residual confounding by familial cardiovascular risk factors is a likely explanation for the association between death of a parent during childhood and hypertension. A family history of early CVD is a well-established

For peer review only - http://bmjopen.bmj.com/site/about/guidelines.xhtml

strong risk factor of hypertension in children and youth.[51] An alternative explanation may be that severe stress increases the risk of hypertension in youth only among individuals with a genetic susceptibility to cardiometabolic disorders; children exposed to parental death due to natural causes are more likely to have such a susceptibility than children who lost a parent due to unnatural causes. A further though – in light of the fact that exposure in young age was not associated with the outcome – less likely explanation for the association between parental death due to natural causes and the increased risk of hypertension is that natural deaths may be proceeded by a long period of disease which may induce chronic stress for family members.[52] The stronger association between death of a parent in adolescence and hypertension than in earlier periods of life – besides better statistical power – is also likely to be due to residual confounding, given the higher proportion of parental deaths related to cardiometabolic conditions in the older age group.

A possible explanation for the limited evidence for an association between the death of a parent and BP in our study, in contrast to the large body of evidence documenting a link between (1) psychological stress and hypertension in adulthood[53] and (2) childhood adversities and hypertension in middle- or old age[28-30] may be related to the age of the BP assessment. Though children who lost a parent may experience chronic stress, the accumulation of further childhood (e.g. adverse socioeconomic circumstances, low social support, abuse or neglect, poor mental health) or adult adversities (e.g. low educational attainment, difficulties in attaching to a partner, difficulties on the labor marker) and the subsequent allostatic load may need to be chronic and act for a longer period than what we studied to induce abnormal changes in BP.[54] At the age of 18-20 years differences in BP may still be small across individuals.[34] Allostatic load may need to interact with the age-related vulnerability to increase BP later in life. This hypotheses is supported by findings of Su and associates who assessed BP on average 13 times between the age of 5 and 38 years, and found that mean SBP and DBP were similar in the first two decades

For peer review only - http://bmjopen.bmj.com/site/about/guidelines.xhtml

of life among groups exposed and unexposed to adverse childhood experiences, but from the third decade of life differences among exposure groups in levels of BP became evident and with time increasingly important; the increase in mean BP was steeper with a higher number of adverse childhood experiences.[34]

Our findings need to be considered also in light of the several limitations the study had. First, since only one or two BP measurements were taken at conscription, we did not have the possibility to consider intra-individual variations in BP,[55] neither to reduce an eventual bias related to white-coat hypertension. Nevertheless, several measurements or ambulatory BP monitoring[15 56] may not be feasible in large epidemiological studies such as ours, as these measures may limit the sample size and may generate selection bias, e.g., children with parental death and/or hypertension may be more or less likely to participate in such a study compared to others. The potential misclassification introduced by the single measurement in our study is likely to be non-differential and may eventually result in an underestimation of the true effect. Nevertheless, the BP measurement at conscription followed the standard measurement for screening for hypertension in primary care. Though one measurement of BP is not sufficient to confirm a clinical diagnosis of hypertension,[57] it has a screening value as the BP measure in this cohort has been reported to be an important predictor of later CVD and mortality.[37 58] Second, since the Conscription Cohort included only men, it is not clear whether the results can be generalized to women. Though women may be more sensitive to psychological stress with respect to hypertension than men,[59] earlier studies in this field investigating the association between adverse childhood experiences and youth BP have mixed results in term of gender differences.[34 60 61] Similarly, it is not clear to what extent our findings may be generalizable to the current Swedish young adults or to populations with different risk factor profile, support for bereaved children and healthcare.[62] Third, though we adjusted for a large number of potential covariates, we did not have information on other potential confounders such as diet, living

For peer review only - http://bmjopen.bmj.com/site/about/guidelines.xhtml

environment, and genetic factors. However, we also studied the association between unnatural parental deaths, which is less likely to be affected by cardiovascular risk factors shared by family members, and BP in youth.

In conclusion, we found no evidence that death of a parent was associated with continuous SBP and DBP measured at the age of 18-20. The fact that the association between loss of a parent and the modest risk of hypertension was observed in case of parental deaths due to natural causes but not in case of unnatural deaths, may be indicative of residual confounding by genetic and environmental factors shared by family members.

Author affiliations

[1]Department of Global Public Health, Karolinska Institutet, Stockholm, Sweden

[2]Department of Public Health Sciences, Stockholm University, Stockholm, Sweden

[3]Institute of Environmental Medicine, Karolinska Institutet, Stockholm, Sweden

[4]Department of Public Health and General Practice, Faculty of Medicine, Norwegian University of Science and Technology, Trondheim, Norway

[5]Centre for Health Equity Studies, Stockholm University and Karolinska Institutet, Stockholm, Sweden

[6]Centre for Epidemiology and Community Medicine, Stockholm County Council, Stockholm, Sweden

[7]Statistical Office, Capital Institute of Pediatrics, Beijing, China

Contributors Conceptualization: KDL. Data curation: HC, TH and KDL. Formal analysis: HC. Funding acquisition: KDL, IJ and HC. Methodology: all authors. Resources: TH and KDL. Supervision: KDL, TH, MR, and YF. Writing – original draft: HC. Writing – review and editing: all authors.

For peer review only - http://bmjopen.bmj.com/site/about/guidelines.xhtml

Funding The study was supported by the Swedish Council for Working Life and Social Research (grand number: 2015-00837), by Karolinska Institutet's Research Foundation (grand number: 2018-01924) and by the China Scholarship Council.

Competing interests None declared.

Ethics approval The study was approved by the Regional Ethics Board in Stockholm (reference numbers: 2004-693/5, 2008/323-32 and 2010/604-32).

Patient and public involvement Patients and/or the public were not involved in the design, conduct, reporting or dissemination plans of this research.

Patient consent for publication Not required.

Data sharing statement Data may be obtained from a third party and are not publicly available.

REFERENCES

1. Breslau N, Wilcox HC, Storr CL*, et al.* Trauma exposure and posttraumatic stress disorder: a study of youths in urban America. *Journal of urban health : bulletin of the New York Academy of Medicine* 2004;81:530-44.

2. Li J, Vestergaard M, Cnattingius S*, et al.* Mortality after parental death in childhood: a nationwide cohort study from three Nordic countries. *PLoS medicine* 2014;11:e1001679.

3. Hjern A, Berg L, Arat A*, et al.* Children as next of kin in Sweden. BSA-rapport. Kalmar, 2017:28.

4. Schooling CM, Jiang C, Lam TH*, et al.* Parental death during childhood and adult cardiovascular risk in a developing country: the Guangzhou Biobank Cohort Study. *PloS one* 2011;6:e19675.

5. Sherr L, Croome N, Clucas C*, et al.* Differential effects of single and double parental death on child emotional functioning and daily life in South Africa. *Child welfare* 2014;93:149-72.

6. Dowdney L. Childhood bereavement following parental death. *Journal of child psychology and psychiatry, and allied disciplines* 2000;41:819-30.

For peer review only - http://bmjopen.bmj.com/site/about/guidelines.xhtml

7. Luecken LJ. Childhood attachment and loss experiences affect adult cardiovascular and cortisol function. *Psychosomatic medicine* 1998;60:765-72.

8. Berg L, Rostila M, Saarela J*, et al.* Parental death during childhood and subsequent school performance. *Pediatrics* 2014;133:682-9.

9. Geulayov G, Gunnell D, Holmen TL*, et al.* The association of parental fatal and non-fatal suicidal behaviour with offspring suicidal behaviour and depression: a systematic review and meta-analysis. *Psychological medicine* 2012;42:1567-80.

10. Berg L, Rostila M, Hjern A. Parental death during childhood and depression in young adults - a national cohort study. *Journal of child psychology and psychiatry, and allied disciplines* 2016;57:1092-8.

11. Hamdan S, Mazariegos D, Melhem NM*, et al.* Effect of parental bereavement on health risk behaviors in youth: a 3-year follow-up. *Archives of pediatrics & adolescent medicine* 2012;166:216-23.

12. Hamdan S, Melhem NM, Porta G*, et al.* Alcohol and substance abuse in parentally bereaved youth. *The Journal of clinical psychiatry* 2013;74:828-33.

13. Oliveira AJ, Rostila M, Saarela J*, et al.* The influence of bereavement on body mass index: results from a national Swedish survey. *PLoS One* 2014;9:e95201.

14. Alciati A, Gesuele F, Casazza G*, et al.* The relationship between childhood parental loss and metabolic syndrome in obese subjects. *Stress and health : journal of the International Society for the Investigation of Stress* 2013;29:5-13.

15. Luecken LJ, Kraft A, Appelhans BM*, et al.* Emotional and cardiovascular sensitization to daily stress following childhood parental loss. *Dev Psychol* 2009;45:296-302.

16. Anderson EL, Caleyachetty R, Stafford M*, et al.* Prospective associations of psychosocial adversity in childhood with risk factors for cardiovascular disease in adulthood: the MRC National Survey of Health and Development. *International journal for equity in health* 2017;16:170.

17. Stein DJ, Scott K, Haro Abad JM*, et al.* Early childhood adversity and later hypertension: data from the World Mental Health Survey. *Annals of clinical psychiatry : official journal of the American Academy of Clinical Psychiatrists* 2010;22:19-28.

For peer review only

For peer review only - http://bmjopen.bmj.com/site/about/guidelines.xhtml

18. Chen X, Wang Y. Tracking of blood pressure from childhood to adulthood: a systematic review and meta-regression analysis. *Circulation* 2008;117:3171-80.

19. Merai R, Siegel C, Rakotz M*, et al.* CDC grand rounds: A public health approach to detect and control hypertension. *Morbidity and Mortality Weekly Report* 2016;65:1261-64.

20. Falkner B, DeLoach S, Keith SW*, et al.* High risk blood pressure and obesity increase the risk for left ventricular hypertrophy in African-American adolescents. *The Journal of pediatrics* 2013;162:94-100.

21. Sun SS, Grave GD, Siervogel RM*, et al.* Systolic blood pressure in childhood predicts hypertension and metabolic syndrome later in life. *Pediatrics* 2007;119:237-46.

22. Rovio SP, Pahkala K, Nevalainen J*, et al.* Cardiovascular Risk Factors From Childhood and Midlife Cognitive Performance: The Young Finns Study. *Journal of the American College of Cardiology* 2017;69:2279-89.

23. Sundin PO, Udumyan R, Sjostrom P*, et al.* Predictors in adolescence of ESRD in middle-aged men. *American journal of kidney diseases : the official journal of the National Kidney Foundation* 2014;64:723-9.

24. Franklin BA, Chaddha A. Resting Heart Rate and Blood Pressure as Indices of Cardiovascular and Mortality Risk: IS LOWER INVARIABLY BETTER? *Journal of cardiopulmonary rehabilitation and prevention* 2018;38:353-57.

25. Lim SS, Vos T, Flaxman AD*, et al.* A comparative risk assessment of burden of disease and injury attributable to 67 risk factors and risk factor clusters in 21 regions, 1990-2010: a systematic analysis for the Global Burden of Disease Study 2010. *Lancet (London, England)* 2012;380:2224-60.

26. Kit BK, Kuklina E, Carroll MD*, et al.* Prevalence of and trends in dyslipidemia and blood pressure among US children and adolescents, 1999-2012. *JAMA pediatrics* 2015;169:272-9.

27. Noubiap JJ, Essouma M, Bigna JJ*, et al.* Prevalence of elevated blood pressure in children and adolescents in Africa: a systematic review and meta-analysis. *The Lancet Public health* 2017;2:e375-e86.

For peer review only

28. Springer KW, Sheridan J, Kuo D, *et al.* Long-term physical and mental health consequences of childhood physical abuse: results from a large population-based sample of men and women. *Child abuse & neglect* 2007;31:517-30.

29. Parrish C, Surkan PJ, Martins SS, *et al.* Childhood adversity and adult onset of hypertension and heart disease in Sao Paulo, Brazil. *Preventing chronic disease* 2013;10:E205.

30. Obi IE, McPherson KC, Pollock JS. Childhood adversity and mechanistic links to hypertension risk in adulthood. *British journal of pharmacology* 2019;176:1932-50.

31. Gooding HC, Milliren CE, Austin SB, *et al.* Child Abuse, Resting Blood Pressure, and Blood Pressure Reactivity to Psychosocial Stress. *Journal of pediatric psychology* 2016;41:5-14.

32. Doom JR, Mason SM, Suglia SF, *et al.* Pathways between childhood/adolescent adversity, adolescent socioeconomic status, and long-term cardiovascular disease risk in young adulthood. *Social science & medicine (1982)* 2017;188:166-75.

33. Su S, Wang X, Kapuku GK, *et al.* Adverse childhood experiences are associated with detrimental hemodynamics and elevated circulating endothelin-1 in adolescents and young adults. *Hypertension (Dallas, Tex : 1979)* 2014;64:201-7.

34. Su S, Wang X, Pollock JS, *et al.* Adverse childhood experiences and blood pressure trajectories from childhood to young adulthood: the Georgia stress and Heart study. *Circulation* 2015;131:1674-81.

35. Falkstedt D, Hemmingsson T, Rasmussen F, *et al.* Body mass index in late adolescence and its association with coronary heart disease and stroke in middle age among Swedish men. *International journal of obesity (2005)* 2007;31:777-83.

36. Lindgren M, Robertson J, Adiels M, *et al.* Resting heart rate in late adolescence and long term risk of cardiovascular disease in Swedish men. *International journal of cardiology* 2018;259:109-15.

37. Sundstrom J, Neovius M, Tynelius P, *et al.* Association of blood pressure in late adolescence with subsequent mortality: cohort study of Swedish male conscripts. *BMJ (Clinical research ed)* 2011;342:d643.

38. Britton KA, Gaziano JM, Djousse L. Normal systolic blood pressure and risk of heart failure in US male physicians. *European journal of heart failure* 2009;11:1129-34.

For peer review only - http://bmjopen.bmj.com/site/about/guidelines.xhtml

39. Muntner P, Shimbo D, Carey RM*, et al.* Measurement of Blood Pressure in Humans: A Scientific Statement From the American Heart Association. *Hypertension (Dallas, Tex : 1979)* 2019;73:e35-e66.

40. Flint AC, Conell C, Ren X*, et al.* Effect of Systolic and Diastolic Blood Pressure on Cardiovascular Outcomes. *The New England journal of medicine* 2019;381:243-51.

41. Whelton Pk Fau - Carey RM, Carey Rm Fau - Aronow WS, Aronow Ws Fau - Casey DE, Jr.*, et al.* 2017 ACC/AHA/AAPA/ABC/ACPM/AGS/APhA/ASH/ASPC/NMA/PCNA Guideline for the Prevention, Detection, Evaluation, and Management of High Blood Pressure in Adults: A Report of the American College of Cardiology/American Heart Association Task Force on Clinical Practice Guidelines.

42. Falkstedt D, Lundberg I, Hemmingsson T. Childhood socio-economic position and risk of coronary heart disease in middle age: a study of 49,321 male conscripts. *European journal of public health* 2011;21:713-8.

43. Larsson D, Hemmingsson T, Allebeck P*, et al.* Self-rated health and mortality among young men: what is the relation and how may it be explained? *Scandinavian journal of public health* 2002;30:259-66.

44. Rostila M, Berg L, Saarela J*, et al.* Experience of Sibling Death in Childhood and Risk of Death in Adulthood: A National Cohort Study From Sweden. *American journal of epidemiology* 2017;185:1247-54.

45. Brunner E. Stress mechanisms in coronary heart disease. 2002

46. Stroebe M, Schut H, Stroebe W. Health outcomes of bereavement. *Lancet (London, England)* 2007;370:1960-73.

47. Fuchs A, Mohler E, Reck C*, et al.* The Early Mother-to-Child Bond and Its Unique Prospective Contribution to Child Behavior Evaluated by Mothers and Teachers. *Psychopathology* 2016;49:211-16.

48. Kirwin KM, Hamrin V. Decreasing the risk of complicated bereavement and future psychiatric disorders in children. *Journal of child and adolescent psychiatric nursing : official publication of the Association of Child and Adolescent Psychiatric Nurses, Inc* 2005;18:62-78.

For peer review only

49. Koehn AJ, Kerns KA. Parent-child attachment: meta-analysis of associations with parenting behaviors in middle childhood and adolescence. *Attachment & human development* 2018;20:378-405.

50. Pabayo R, Molnar BE, Kawachi I. Witnessing a violent death and smoking, alcohol consumption, and marijuana use among adolescents. *Journal of urban health : bulletin of the New York Academy of Medicine* 2014;91:335-54.

51. Kawabe H, Azegami T, Takeda A*, et al.* Features of and preventive measures against hypertension in the young.

52. Chen R, Fall K, Czene K*, et al.* Impact of parental cancer on IQ, stress resilience, and physical fitness in young men. *Clinical epidemiology* 2018;10:593-604.

53. Liu MY, Li N, Li WA*, et al.* Association between psychosocial stress and hypertension: a systematic review and meta-analysis.

54. Kuh D, Ben-Shlomo Y. A life course approach to chronic disease epidemiology. 2 ed. Oxford: Oxford University Press 2004.

55. Islam MS. Hypertension: From Basic Research to Clinical Practice. *Advances in experimental medicine and biology* 2017;956:1-2.

56. Buckley T, Mihailidou AS, Bartrop R*, et al.* Haemodynamic changes during early bereavement: potential contribution to increased cardiovascular risk. *Heart, lung & circulation* 2011;20:91-8.

57. Flynn JT, Kaelber DC, Baker-Smith CM*, et al.* Clinical Practice Guideline for Screening and Management of High Blood Pressure in Children and Adolescents. *Pediatrics* 2017;140

58. Falkstedt D, Koupil I, Hemmingsson T. Blood pressure in late adolescence and early incidence of coronary heart disease and stroke in the Swedish 1969 conscription cohort. *Journal of hypertension* 2008;26:1313-20.

59. Di Pilla M, Bruno RM, Taddei S*, et al.* Gender differences in the relationships between psychosocial factors and hypertension. *Maturitas* 2016;93:58-64.

60. Dietz LJ, Pham S, Melhem N*, et al.* Blood pressure recovery to social stress in parentally bereaved and non-bereaved youths. *Journal of psychosomatic research* 2018;113:58-65.

For peer review only

61. Luecken LJ, Roubinov DS. Hostile behavior links negative childhood family relationships to heart rate reactivity and recovery in young adulthood. *International journal of psychophysiology : official journal of the International Organization of Psychophysiology* 2012;84:172-9.

62. Rostila M. Commentary: Childhood parental loss and adulthood health: discussing the role of parental cause of death, child's age at death and historical context. *Social science & medicine (1982)* 2015;131:190-2.

For peer review only

For peer review only - http://bmjopen.bmj.com/site/about/guidelines.xhtml

Table1. Characteristics of study participants according to death of a parent (N=48624)

Variables	Total, n (%)	Death of a parent		*P*-value*
		Yes (n=3504)	No (n=45120)	
Categorical variables, n (%)				
Parental socioeconomic status				<0.01
Middle or high level non-manual employee	10716 (22.0)	689 (19.7)	10027 (22.2)	
Low level non-manual employee	4942 (10.2)	300 (8.6)	4642 (10.3)	
Skilled worker	10403 (21.4)	510 (14.6)	9893 (21.9)	
Unskilled worker	16094 (33.1)	1121 (32.0)	14973 (33.2)	
Farmer	5373 (11.0)	399 (11.4)	4974 (11.0)	
Other	1096 (2.3)	485 (13.8)	611 (1.4)	
Depression				<0.01
No	47892 (98.5)	3421 (97.6)	44471 (98.6)	
Yes	732 (1.5)	83 (2.4)	649 (1.4)	
Anxiety				0.72
No	48469 (99.7)	3494 (99.7)	44975 (99.7)	
Yes	155 (0.3)	10 (0.3)	145 (0.3)	
Cardiorespiratory fitness (stanine scores)				<0.01
1	34 (0.1)	2 (0.1)	32 (0.1)	
2	207 (0.4)	15 (0.4)	192 (0.4)	
3	2451 (5.0)	207 (5.9)	2244 (5.0)	
4	7020 (14.4)	578 (16.5)	6442 (14.3)	
5	11447 (23.5)	857 (24.4)	10590 (23.5)	

For peer review only - http://bmjopen.bmj.com/site/about/guidelines.xhtml

6	9215 (19.0)	658 (18.8)	8557 (19.0)	
7	5713 (11.8)	401 (11.4)	5312 (11.8)	
8	4608 (9.5)	305 (8.7)	4303 (9.5)	
9	7867 (16.2)	480 (13.7)	7387 (16.4)	
Missing	62 (0.1)	1 (0.1)	61 (0.1)	
Number of personal friends				0.72
0	389 (0.8)	29 (0.8)	360 (0.8)	
1-3	3260 (6.7)	240 (6.8)	3020 (6.7)	
3-5	15195 (31.3)	1114 (31.8)	14081 (31.2)	
>5	29006 (59.6)	2048 (58.4)	26958 (59.7)	
Missing	774 (1.6)	73 (2.1)	701 (1.6)	
Confidential discussions with friends				<0.01
Never	2011 (4.2)	178 (5.1)	1833 (4.1)	
Sometime	28655 (58.9)	2061 (58.8)	26594 (58.9)	
Quite often	17072 (35.1)	1186 (33.8)	15886 (35.2)	
Missing	886 (1.8)	79 (2.3)	807 (1.8)	
Has a confidant				<0.01
No	7865 (16.2)	633 (18.1)	7232 (16.0)	
Yes	39741 (81.7)	2772 (79.1)	36969 (81.9)	
Missing	1018 (2.1)	99 (2.8)	919 (2.0)	
Number of cigarettes smoked per day				
0	19903 (40.9)	1216 (34.7)	18687 (41.4)	<0.01
1-10	15310 (31.5)	1150 (32.8)	14160 (31.4)	
>10	12731 (26.2)	1067 (30.5)	11664 (25.9)	
Missing	680 (1.4)	71 (2.0)	609 (1.3)	

For peer review only

For peer review only - http://bmjopen.bmj.com/site/about/guidelines.xhtml

Risky drinking behavior				<0.01
No	42323 (87.0)	2923 (83.4)	39400 (87.3)	
Yes	6267 (12.9)	579 (16.5)	5688 (12.6)	
Missing	34 (0.1)	2 (0.1)	32 (0.1)	
Father's drinking habits				<0.01
Never, occasionally or sometimes	45580 (93.8)	3048 (87.0)	42532 (94.3)	
Often	1954 (4.0)	175 (5.0)	1779 (3.9)	
Missing	1090 (2.2)	281 (8.0)	809 (1.8)	
Cumulative adverse childhood experiences				<0.01
0	9617 (19.8)	482 (13.8)	9135 (20.2)	
1	18477 (38.0)	1043 (29.8)	17434 (38.6)	
2	12173 (25.0)	1061 (30.3)	11112 (24.6)	
⩾3	8357 (17.2)	918 (26.2)	7439 (16.5)	
Continuous variable, median				
Body mass index, kg/m^2	20.6	20.6	20.7	0.71

*Men with no missing data on the corresponding variables were included in these analyses.

For peer review only

For peer review only - http://bmjopen.bmj.com/site/about/guidelines.xhtml

Table 2. Least square means and 95% confidence intervals for blood pressure according to exposure to death of a parent during childhood (N=48624)

Type of exposure	Model 1*		Model 2†		Model 3‡	
	LS Mean (95% CI)	*P*-value	LS Mean (95% CI)	*P*-value	LS Mean (95% CI)	*P*-value
**Systolic blood pressure, mmHg**						
Unexposed	126.09 (125.99-126.20)	-	126.13 (125.97-126.29)	-	128.02 (126.86-129.18)	-
Any loss	126.34 (125.95-126.72)	0.24	126.35 (125.96-126.74)	0.30	128.25 (127.04-129.46)	0.26
Cause of death of the parent§						
Unnatural death	125.49 (124.58-126.40)	0.20	125.56 (124.65-126.47)	0.23	127.57 (126.10-129.03)	0.59
Cardiovascular death	126.94 (126.01-127.87)	0.08	126.99 (126.06-127.92)	0.07	128.40 (126.92-129.88)	0.22
Other natural cause	126.62 (126.05-127.19)	0.08	126.59 (126.02-127.16)	0.12	128.31 (127.02-129.59)	0.10
Gender of deceased parent§						
Mother	126.36 (125.59-127.14)	0.50	126.26 (125.47-127.04)	0.74	128.14 (126.74-129.53)	0.51
Father	126.50 (126.00-126.99)	0.12	126.55 (126.06-127.05)	0.10	128.30 (127.04-129.55)	0.11
Child's age at loss						
≤5 years	125.93 (125.24-126.62)	0.65	125.96 (125.26-126.65)	0.60	127.87 (126.54-129.20)	0.63

For peer review only - http://bmjopen.bmj.com/site/about/guidelines.xhtml

6-12 years	126.30 (125.57-127.04)	0.58	126.30 (125.56-127.03)	0.69	128.15 (126.79-129.51)	0.77
13-18 years	126.67 (126.06-127.28)	0.07	126.69 (126.08-127.31)	0.08	128.64 (127.34-129.94)	0.05
**Diastolic blood pressure, mmHg**						
Unexposed	72.90 (72.82-72.99)	-	72.94 (72.81-73.07)		73.99 (73.06-74.93)	-
Any loss	72.88 (72.57-73.19)	0.87	72.86 (72.55-73.17)	0.63	73.86 (72.89-74.84)	0.45
Cause of death of the parent§						
Unnatural death	72.16 (71.43-72.88)	0.05	72.10 (71.37-72.82)	0.02	73.20 (72.02-74.39)	0.06
Cardiovascular death	73.42 (72.68-74.16)	0.18	73.40 (72.66-74.14)	0.24	74.14 (72.94-75.33)	0.60
Other natural cause	73.13 (72.67-73.58)	0.34	73.12 (72.67-73.58)	0.46	74.14 (73.10-75.18)	0.40
Gender of deceased parent§						
Mother	72.36 (71.74-72.97)	0.08	72.39 (71.76-73.01)	0.09	73.43 (72.31-74.56)	0.16
Father	73.16 (72.77-73.55)	0.20	73.11 (72.72-73.51)	0.38	74.00 (72.99-75.01)	0.58
Child's age at loss						
≤5 years	72.62 (72.07-73.17)	0.32	72.57 (72.02-73.12)	0.20	73.49 (72.42-74.57)	0.08
6-12 years	73.04 (72.46-73.62)	0.66	72.99 (72.41-73.57)	0.88	73.98 (72.88-75.08)	0.93
13-18 years	72.97 (72.49-73.45)	0.80	72.99 (72.50-73.49)	0.84	74.09 (73.04-75.14)	0.74

For peer review only - http://bmjopen.bmj.com/site/about/guidelines.xhtml

LS Mean=least square mean; CI=confidence intervals.

*Model 1 was unadjusted.

†Model 2 was adjusted for parental socioeconomic status.

‡Model 3 was adjusted for parental socioeconomic status, depression, anxiety, body-mass index, cardiorespiratory fitness, number of friends, frequency of talking with friends confidentially, having a confidant, number of cigarettes smoked per day, and risky drinking behavior.

§Only men with no missing data on this type of exposure were included.

For peer review only - http://bmjopen.bmj.com/site/about/guidelines.xhtml

Table 3. Least square means and 95% confidence intervals for systolic blood pressure and diastolic blood pressure, by gender of the deceased parent during childhood and further classified according to the cause of death and the age at loss (N=48624)

Type of exposure	Model 1*		Model 2†		Model 3‡	
	LS Mean (95% CI)	*P*-value	LS Mean (95% CI)	*P*-value	LS Mean (95% CI)	*P*-value
**Systolic blood pressure, mmHg**						
Unexposed	126.09 (125.99-126.20)	-	126.12 (125.96-126.28)	-	127.81 (126.65-128.98)	-
Death of a mother§						
Cause of death						
Unnatural death	125.35 (123.05-127.66)	0.53	125.57 (123.27-127.87)	0.64	127.43 (124.84-130.02)	0.75
Cardiovascular death	126.42 (123.84-129.00)	0.81	126.30 (123.73-128.87)	0.89	127.87 (125.00-130.73)	0.97
Other natural death	126.62 (125.72-127.51)	0.25	126.47 (125.57-127.37)	0.44	128.27 (126.81-129.73)	0.32
Child's age at loss						
≤5 years	124.99 (123.15-126.84)	0.24	124.75 (122.91-126.59)	0.14	126.55 (124.36-128.74)	0.16
6-12 years	126.29 (125.01-127.56)	0.77	126.16 (124.89-127.44)	0.95	127.79 (126.07-129.51)	0.91
13-18 years	126.95 (125.80-128.10)	0.14	126.91 (125.76-128.07)	0.18	129.00 (127.37-130.63)	0.05
Death of a father§						

For peer review only - http://bmjopen.bmj.com/site/about/guidelines.xhtml

Cause of death						
Unnatural death	125.51 (124.52-126.51)	0.25	125.56 (124.57-126.55)	0.27	127.59 (126.07-129.11)	0.66
Cardiovascular death	127.02 (126.02-128.02)	0.07	127.10 (126.10-128.09)	0.06	128.47 (126.95-129.99)	0.20
Other natural death	126.62 (125.88-127.37)	0.17	126.68 (125.93-127.42)	0.15	128.33 (126.96-129.70)	0.18
Child's age at loss						
≤5 years	126.60 (125.58-127.63)	0.33	126.74 (125.70-127.77)	0.26	128.55 (127.01-130.09)	0.21
6-12 years	126.31 (125.41-127.20)	0.64	126.38 (125.48-127.28)	0.59	128.13 (126.66-129.59)	0.59
13-18 years	126.56 (125.85-127.28)	0.20	126.58 (125.86-127.30)	0.22	128.28 (126.92-129.63)	0.27
**Diastolic blood pressure, mmHg**						
Unexposed	72.90 (72.82-72.99)	-	72.93 (72.80-73.06)	-	73.92 (72.98-74.86)	-
Death of a mother§						
Cause of death						
Unnatural death	70.68 (68.84-72.51)	0.02	70.67 (68.84-72.51)	0.02	72.28 (70.18-74.37)	0.09
Cardiovascular death	72.09 (70.04-74.14)	0.44	72.13 (70.07-74.18)	0.44	72.61 (70.29-74.92)	0.22
Other natural death	72.69 (71.98-73.40)	0.55	72.73 (72.01-73.45)	0.58	73.81 (72.63-74.99)	0.77
Child's age at loss						

For peer review only - http://bmjopen.bmj.com/site/about/guidelines.xhtml

≤5 years	71.83 (70.37-73.30)	0.15	71.86 (70.40-73.33)	0.16	74.35 (73.12-75.58)	0.08
6-12 years	72.16 (71.14-73.17)	0.15	72.20 (71.18-73.22)	0.16	74.35 (73.25-75.46)	0.17
13-18 years	72.72 (71.80-73.63)	0.69	72.74 (71.82-73.66)	0.69	73.37 (72.14-74.60)	0.81
Death of a father§						
Cause of death						
Unnatural death	72.43 (71.64-73.22)	0.24	72.36 (71.57-73.15)	0.17	74.00 (72.69-75.31)	0.18
Cardiovascular death	73.62 (72.82-74.41)	0.08	73.59 (72.79-74.38)	0.11	72.52 (70.75-74.29)	0.30
Other natural death	73.44 (72.85-74.03)	0.08	73.39 (72.80-73.99)	0.14	73.16 (71.77-74.55)	0.17
Child's age at loss						
≤5 years	72.96 (72.14-73.78)	0.90	72.84 (72.01-73.67)	0.84	73.76 (72.52-75.01)	0.77
6-12 years	73.47 (72.76-74.19)	0.12	73.39 (72.67-74.11)	0.22	74.25 (73.07-75.43)	0.34
13-18 years	73.07 (72.50-73.64)	0.58	73.07 (72.50-73.65)	0.62	73.96 (72.87-75.06)	0.79

LS Mean=least square mean; CI=confidence intervals.

*Model 1 was unadjusted.

†Model 2 was adjusted for parental socioeconomic status.

For peer review only - http://bmjopen.bmj.com/site/about/guidelines.xhtml

‡Model 3 was adjusted for parental socioeconomic status, depression, anxiety, body-mass index, cardiorespiratory fitness, number of friends, frequency of talking with friends confidentially, having a confidant, number of cigarettes smoked per day, and risky drinking behavior.

§Only men without missing data on type of exposure were include

For peer review only

For peer review only - http://bmjopen.bmj.com/site/about/guidelines.xhtml

Table 4. Odds ratios for high blood pressure at conscription according to exposure to death of a parent during childhood (N=48624)

Type of exposure	Event	Model 1*	Model 2†	Model 3‡
		OR (95% CI)	OR (95% CI)	OR (95% CI)
Unexposed	9166	Ref.	Ref.	Ref.
Any loss	776	1.12 (1.03-1.21)	1.11 (1.02-1.21)	1.10 (1.00-1.20)
Cause of death of the parent§				
Unnatural death	119	0.91 (0.74-1.11)	0.91 (0.74-1.11)	0.93 (0.75-1.15)
Cardiovascular death	142	1.20 (0.99-1.45)	1.20 (0.99-1.45)	1.15 (0.95-1.40)
Other natural cause	383	1.23 (1.09-1.38)	1.21 (1.08-1.37)	1.20 (1.06-1.35)
Child's age at loss				
≤5 years	218	0.97 (0.83-1.13)	0.96 (0.82-1.12)	0.94 (0.81-1.11)
6-12 years	226	1.18 (1.01-1.37)	1.16 (1.00-1.36)	1.15 (0.98-1.35)
13-18 years	332	1.19 (1.05-1.35)	1.19 (1.05-1.35)	1.18 (1.03-1.34)
Gender of deceased parent§				
Mother	196	1.13 (0.96-1.33)	1.11 (0.95-1.30)	1.12 (0.94-1.32)
Cause of mother's death				
Unnatural death	21	1.06 (0.65-1.71)	1.08 (0.67-1.76)	1.06 (0.64-1.77)
Cardiovascular death	14	0.84 (0.47-1.51)	0.83 (0.46-1.48)	0.88 (0.49-1.60)
Other natural death	155	1.20 (1.00-1.44)	1.18 (0.98-1.41)	1.18 (0.98-1.42)
Child's age at mother's death				
≤5 years	25	0.75 (0.49-1.16)	0.73 (0.47-1.12)	0.65 (0.41-1.04)
6-12 years	81	1.31 (1.02-1.69)	1.29 (1.00-1.66)	1.27 (0.97-1.65)
13-18 years	90	1.15 (0.91-1.45)	1.14 (0.90-1.44)	1.19 (0.93-1.52)
Father	493	1.15 (1.03-1.27)	1.15 (1.03-1.28)	1.13 (1.01-1.26)

For peer review only - http://bmjopen.bmj.com/site/about/guidelines.xhtml

Cause of father's death				
Unnatural death	98	0.88 (0.71-1.10)	0.88 (0.70-1.10)	0.91 (0.72-1.14)
Cardiovascular death	128	1.26 (1.03-1.54)	1.26 (1.03-1.54)	1.19 (0.97-1.47)
Other natural death	228	1.24 (1.07-1.44)	1.24 (1.06-1.45)	1.21 (1.03-1.42)
Child's age at mother's death				
≤5 years	106	1.06 (0.85-1.32)	1.06 (0.85-1.33)	1.06 (0.84-1.33)
6-12 years	145	1.11 (0.92-1.34)	1.11 (0.92-1.34)	1.10 (0.90-1.34)
13-18 years	242	1.21 (1.05-1.40)	1.21 (1.05-1.40)	1.17 (1.01-1.37)
Child's age at loss§				
≤5 years				
Cause of parent's death				
Unnatural death	17	0.61 (0.37-1.02)	0.61 (0.36-1.02)	0.61 (0.36-1.05)
Cardiovascular death	12	0.96 (0.51-1.81)	0.97 (0.52-1.84)	1.01 (0.53-1.93)
Other natural death	57	1.11 (0.83-1.49)	1.09 (0.81-1.47)	1.04 (0.76-1.41)
6-12 years				
Cause of parent's death				
Unnatural death	41	0.86 (0.61-1.20)	0.84 (0.60-1.19)	0.85 (0.59-1.21)
Cardiovascular death	46	1.30 (0.93-1.81)	1.29 (0.92-1.81)	1.24 (0.88-1.77)
Other natural death	139	1.28 (1.06-1.55)	1.26 (1.04-1.53)	1.24 (1.01-1.52)
13-18 years				
Cause of parent's death				
Unnatural death	61	1.10 (0.83-1.46)	1.11 (0.84-1.48)	1.15 (0.86-1.54)
Cardiovascular death	84	1.19 (0.93-1.53)	1.19 (0.93-1.52)	1.12 (0.87-1.45)
Other natural death	187	1.23 (1.04-1.45)	1.22 (1.03-1.44)	1.21 (1.02-1.44)

OR=odds ratio, CI=confidence intervals.

For peer review only - http://bmjopen.bmj.com/site/about/guidelines.xhtml

*Model 1 was unadjusted.

†Model 2 was adjusted for parental socioeconomic status.

‡Model 3 was adjusted for parental socioeconomic status, depression, anxiety, body-mass index, cardiorespiratory fitness, number of friends, frequency of talking with friends confidentially, having a confidant, number of cigarettes smoked per day, and risky drinking behavior.

§Only men with no missing data on this type of exposure were included.

For peer review only

Supplementary table 1. Associations between death of a parent during childhood and least square means for systolic and diastolic blood pressure at conscription after excluding exposed study participants whose exposure status was defined using questionnaire data (N= 47968)

Type of exposure	Model 1*		Model 2†		Model 3‡	
	LS Mean (95% CI)	*P*-value	LS Mean (95% CI)	*P*-value	LS Mean (95% CI)	*P*-value
**Systolic blood pressure**						
Unexposed	126.09 (125.99-126.20)	-	126.12 (125.96-126.28)	-	127.81 (126.65-128.98)	-
Any loss	126.44 (126.01-126.87)	0.13	126.45 (126.02-126.88)	0.15	128.16 (126.93-129.39)	0.13
Cause of death of the parent						
Unnatural death	125.49 (124.58-126.40)	0.20	125.56 (124.65-126.47)	0.23	127.57 (126.10-129.03)	0.59
Cardiovascular death	126.94 (126.01-127.87)	0.08	126.99 (126.06-127.92)	0.07	128.40 (126.92-129.88)	0.22
Other natural cause	126.62 (126.05-127.19)	0.08	126.59 (126.02-127.16)	0.12	128.31 (127.02-129.59)	0.10
Gender of deceased parent						
Mother	126.45 (125.66-127.24)	0.38	126.35 (125.55-127.14)	0.57	128.13 (126.73-129.54)	0.43
Father	126.43 (125.92-126.94)	0.20	126.49 (125.98-127.00)	0.17	128.17 (126.91-129.43)	0.19
Child’s age at loss						
≤5 years	125.98 (124.90-127.07)	0.84	126.04 (124.95-127.13)	0.87	127.75 (126.17-129.33)	0.89

For peer review only

6-12 years	126.30 (125.57-127.03)	0.58	126.30 (125.57-127.03)	0.66	127.96 (126.59-129.33)	0.74
13-18 years	126.67 (126.06-127.28)	0.07	126.68 (126.06-127.30)	0.08	128.44 (127.13-129.75)	0.05
**Diastolic blood pressure**						
Unexposed	72.90 (72.82-72.99)	-	72.94 (72.81-73.07)	-	73.93 (72.99-74.87)	-
Any loss	72.98 (72.63-73.32)	0.70	72.95 (72.61-73.30)	0.95	73.93 (72.93-74.92)	0.99
Cause of death of the parent						
Unnatural death	72.16 (71.43-72.88)	0.05	72.10 (71.37-72.82)	0.02	73.20 (72.02-74.39)	0.06
Cardiovascular death	73.42 (72.68-74.16)	0.18	73.40 (72.66-74.14)	0.24	74.14 (72.94-75.33)	0.60
Other natural cause	73.13 (72.67-73.58)	0.34	73.12 (72.67-73.58)	0.46	74.14 (73.10-75.18)	0.40
Gender of deceased parent						
Mother	72.39 (71.76-73.02)	0.11	72.43 (71.79-73.06)	0.12	73.52 (72.39-74.66)	0.23
Father	73.22 (72.81-73.63)	0.14	73.17 (72.76-73.58)	0.27	74.09 (73.07-75.11)	0.43
Child's age at loss						
≤5 years	72.85 (71.99-73.72)	0.91	72.75 (71.87-73.62)	0.66	73.68 (72.40-74.95)	0.57
6-12 years	73.04 (72.46-73.62)	0.66	72.99 (72.40-73.57)	0.89	73.92 (72.81-75.02)	0.95
13-18 years	72.97 (72.49-73.45)	0.80	73.00 (72.50-73.49)	0.84	74.02 (72.97-75.08)	0.74

For peer review only

LS Mean=least square mean; CI=confidence intervals.

*Model 1 was unadjusted.

†Model 2 was adjusted for parental socioeconomic status.

‡Model 3 was adjusted for parental socioeconomic status, depression, anxiety, body-mass index, cardiorespiratory fitness, number of friends, frequency of talking with friends confidentially, having a confidant, number of cigarettes smoked per day, and risky drinking behavior.

For peer review only

Supplementary table 2. Associations between death of a parent and blood pressure at conscription, stratified by parental socioeconomic status and childhood adversity (N=48624)

Potential effect modifiers	Systolic blood pressure		Diastolic blood pressure	
	LS Mean (95% CI)*	P-value†	LS Mean (95% CI)*	P-value†
Parental socioeconomic status				
Non-manual employee	126.75 (124.57-128.92)	-	73.61 (71.84-75.38)	-
Unskilled worker/Skilled worker	129.11 (127.53-130.70)	0.86	73.94 (72.66-75.22)	0.72
Famer/Other	129.61 (125.66-133.56)	0.64	76.09 (72.92-79.26)	0.01
Cumulative childhood adversity				
0	126.61 (122.78-130.44)	-	75.47 (72.38-78.56)	-
1	130.75 (128.58-132.92)	0.84	74.41 (72.66-76.16)	0.16
2	127.95 (125.56-130.35)	0.68	74.08 (72.16-76.00)	<0.01
≥3	127.37 (125.14-129.60)	0.96	73.00 (71.17-74.83)	0.13

LS Mean=least square mean; CI=confidence intervals.

For peer review only

*Models were adjusted for parental socioeconomic status, depression, anxiety, body-mass index, cardiorespiratory fitness, number of friends, frequency of talking with friends confidentially, having a confidant, number of cigarettes smoked per day, risky drinking behavior and cumulative childhood experience.

†*P*-value for interaction between death of a parent and the potential effect modifier.

For peer review only

STROBE 2007 (v4) Statement—Checklist of items that should be included in reports of *cohort studies*

Section/Topic	Item #	Recommendation	Reported on page #
**Title and abstract**	1	(*a*) Indicate the study's design with a commonly used term in the title or the abstract	1
		(*b*) Provide in the abstract an informative and balanced summary of what was done and what was found	2
**Introduction**			4-5
Background/rationale	2	Explain the scientific background and rationale for the investigation being reported	5
Objectives	3	State specific objectives, including any prespecified hypotheses	
**Methods**			
Study design	4	Present key elements of study design early in the paper	5
Setting	5	Describe the setting, locations, and relevant dates, including periods of recruitment, exposure, follow-up, and data collection	5
Participants	6	(*a*) Give the eligibility criteria, and the sources and methods of selection of participants. Describe methods of follow-up	5
		(*b*) For matched studies, give matching criteria and number of exposed and unexposed	NA
Variables	7	Clearly define all outcomes, exposures, predictors, potential confounders, and effect modifiers. Give diagnostic criteria, if applicable	6-8
Data sources/ measurement	8*	For each variable of interest, give sources of data and details of methods of assessment (measurement). Describe comparability of assessment methods if there is more than one group	6-8
Bias	9	Describe any efforts to address potential sources of bias	5-8
Study size	10	Explain how the study size was arrived at	5
Quantitative variables	11	Explain how quantitative variables were handled in the analyses. If applicable, describe which groupings were chosen and why	6-8
Statistical methods	12	(*a*) Describe all statistical methods, including those used to control for confounding	8-9
		(*b*) Describe any methods used to examine subgroups and interactions	8-9
		(*c*) Explain how missing data were addressed	5, 9
		(*d*) If applicable, explain how loss to follow-up was addressed	5
		(*e*) Describe any sensitivity analyses	8-9
**Results**			

For peer review only

Participants	13*	(a) Report numbers of individuals at each stage of study—eg numbers potentially eligible, examined for eligibility, confirmed eligible, included in the study, completing follow-up, and analysed	NA
		(b) Give reasons for non-participation at each stage	NA
		(c) Consider use of a flow diagram	NA
Descriptive data	14*	(a) Give characteristics of study participants (eg demographic, clinical, social) and information on exposures and potential confounders	9, 24-25
		(b) Indicate number of participants with missing data for each variable of interest	24-25
		(c) Summarise follow-up time (eg, average and total amount)	NA
Outcome data	15*	Report numbers of outcome events or summary measures over time	10
Main results	16	(*a*) Give unadjusted estimates and, if applicable, confounder-adjusted estimates and their precision (eg, 95% confidence interval). Make clear which confounders were adjusted for and why they were included	10, 26-34
		(*b*) Report category boundaries when continuous variables were categorized	10, 26-34
		(*c*) If relevant, consider translating estimates of relative risk into absolute risk for a meaningful time period	NA
Other analyses	17	Report other analyses done—eg analyses of subgroups and interactions, and sensitivity analyses	10
**Discussion**			
Key results	18	Summarise key results with reference to study objectives	11
**Limitations**			
Interpretation	20	Give a cautious overall interpretation of results considering objectives, limitations, multiplicity of analyses, results from similar studies, and other relevant evidence	11-15
Generalisability	21	Discuss the generalisability (external validity) of the study results	14
**Other information**			
Funding	22	Give the source of funding and the role of the funders for the present study and, if applicable, for the original study on which the present article is based	16

*Give information separately for cases and controls in case-control studies and, if applicable, for exposed and unexposed groups in cohort and cross-sectional studies.

Note: An Explanation and Elaboration article discusses each checklist item and gives methodological background and published examples of transparent reporting. The STROBE checklist is best used in conjunction with this article (freely available on the Web sites of PLoS Medicine at http://www.plosmedicine.org/, Annals of Internal Medicine at http://www.annals.org/, and Epidemiology at http://www.epidem.com/). Information on the STROBE Initiative is available at www.strobe-statement.org.

For peer review only

Death of a parent during childhood and blood pressure in youth: a population-based cohort study of Swedish men

Journal:	*BMJ Open*
Manuscript ID	bmjopen-2020-043657.R1
Article Type:	Original research
Date Submitted by the Author:	22-Feb-2021
Complete List of Authors:	Chen, Hua; Karolinska Institutet, Department of Global Public Health<br>Hemmingsson, Tomas; Stockholm University, Department of Public Health Sciences; Karolinska Institutet, Institute of Environmental Medicine<br>Janszky, Imre ; Karolinska Institutet, Department of Global Public Health; Norwegian University of Science and Technology, Department of Public Health and General Practice, Faculty of Medicine<br>Rostila, Mikael ; Stockholm University, Department of Public Health Sciences; Centre for Health Equity Studies, Stockholm University and Karolinska Institutet<br>Forsell , Yvonne; Karolinska Institutet, Department of Global Public Health; Stockholm County Council, Centre for Epidemiology and Community Medicine<br>Meng, Linghui; Karolinska Institutet, Department of Global Public Health; Capital Institute of Pediatrics, Statistical Office<br>Liang, Yajun; Karolinska Institute, Department of Global Public Health<br>Laszlo, Krisztina; Karolinska Institutet, Department of Global Public Health
**Primary Subject Heading**:	Epidemiology
Secondary Subject Heading:	Public health
Keywords:	Hypertension < CARDIOLOGY, EPIDEMIOLOGY, MENTAL HEALTH

SCHOLARONE™
Manuscripts

I, the Submitting Author has the right to grant and does grant on behalf of all authors of the Work (as defined in the below author licence), an exclusive licence and/or a non-exclusive licence for contributions from authors who are: i) UK Crown employees; ii) where BMJ has agreed a CC-BY licence shall apply, and/or iii) in accordance with the terms applicable for US Federal Government officers or employees acting as part of their official duties; on a worldwide, perpetual, irrevocable, royalty-free basis to BMJ Publishing Group Ltd ("BMJ") its licensees and where the relevant Journal is co-owned by BMJ to the co-owners of the Journal, to publish the Work in this journal and any other BMJ products and to exploit all rights, as set out in our licence.

The Submitting Author accepts and understands that any supply made under these terms is made by BMJ to the Submitting Author unless you are acting as an employee on behalf of your employer or a postgraduate student of an affiliated institution which is paying any applicable article publishing charge ("APC") for Open Access articles. Where the Submitting Author wishes to make the Work available on an Open Access basis (and intends to pay the relevant APC), the terms of reuse of such Open Access shall be governed by a Creative Commons licence – details of these licences and which Creative Commons licence will apply to this Work are set out in our licence referred to above.

Other than as permitted in any relevant BMJ Author's Self Archiving Policies, I confirm this Work has not been accepted for publication elsewhere, is not being considered for publication elsewhere and does not duplicate material already published. I confirm all authors consent to publication of this Work and authorise the granting of this licence.

For peer review only

Death of a parent during childhood and blood pressure in youth: a population-based cohort study of Swedish men

Hua Chen,[1]* Tomas Hemmingsson,[2,3] Imre Janszky,[1,4] Mikael Rostila,[2,5] Yvonne Forsell,[1,6] Linghui Meng,[1,7] Yajun Liang,[1] Krisztina D. László[1]

Author affiliations

[1]Department of Global Public Health, Karolinska Institutet, Stockholm, Sweden

[2]Department of Public Health Sciences, Stockholm University, Stockholm, Sweden

[3]Institute of Environmental Medicine, Karolinska Institutet, Stockholm, Sweden

[4]Department of Public Health and General Practice, Faculty of Medicine, Norwegian University of Science and Technology, Trondheim, Norway

[5]Centre for Health Equity Studies, Stockholm University and Karolinska Institutet, Stockholm, Sweden

[6]Centre for Epidemiology and Community Medicine, Stockholm County Council, Stockholm, Sweden

[7]Statistical Office, Capital Institute of Pediatrics, Beijing, China

*Address correspondence to Hua Chen, Department of Global Public Health, Karolinska Institutet, Tomtebodavägen 18A, Stockholm, 171 77, Sweden. E-mail: hua.chen@ki.se

For peer review only

ABSTRACT

Objective Compelling evidence suggests that childhood adversities are associated with an increased risk of hypertension in middle- and old age. The link between childhood adversities and blood pressure in youth is less clear. In this cohort study, we examined the association between death of a parent during childhood and blood pressure in early adulthood in men.

Setting Sweden.

Participants We studied 48,624 men born in 1949-51 who participated in the compulsory military conscription in 1969/70 in Sweden. Information on death of a parent during childhood was obtained from population-based registers. Information on covariates was obtained from the questionnaire and the clinical examination completed at conscription and from population-based registers.

Outcome measures Blood pressure was measured at conscription according to standard procedures.

Results The multivariable least square means of systolic and diastolic blood pressure did not differ between bereaved [128.25 (127.04-129.46) and 73.86 (72.89-74.84) mmHg] and non-bereaved study participants [128.02 (126.86-129.18) and 73.99 (73.06-74.93) mmHg]. Results were similar when considering the cause of the parent's death, the gender of the deceased parent, or the child's age at loss. Loss of a parent in childhood tended to be associated with an increased hypertension risk [odds ratio and 95% confidence interval: 1.10 (1.00-1.20)]; the association was present only in case of natural deaths.

Conclusion We found no strong support for the hypothesis that stress following the loss of a parent during childhood is associated with blood pressure or hypertension in youth in men.

For peer review only - http://bmjopen.bmj.com/site/about/guidelines.xhtml

Strengths and limitations of this study

- This is the first study to investigate prospectively the association between parental death in childhood and blood pressure measured in early adulthood in a large national cohort; the prospective design limited recall bias, while the large national sample allowed to reduce selection bias and to conduct sub-analyses to test hypotheses that may be supportive of a causal effect, if any.
- Since only one or two blood pressure measurements were taken, there was no possibility to consider intra-individual variations in blood pressure or to reduce the impact of white-coat hypertension.
- Information on some potential confounders was not available.
- It is not clear to what extent our findings are generalizable to women, to current Swedish young adults or to populations with different risk factor profiles, support for bereaved children and healthcare.

For peer review only - http://bmjopen.bmj.com/site/about/guidelines.xhtml

INTRODUCTION

Death of a parent is one of the most traumatic and stressful events that a child can experience.[1] In the Nordic countries around 4% of children experience parental death before the age of 18 years,[2 3] while in the developing countries the prevalence is higher.[4 5] Children exposed to parental death have an increased risk of experiencing poor social support,[6] heightened stress reactivity,[7] poor school performance,[8] emotional and behavioral problems,[6] psychiatric disorders,[9 10] substance use,[11 12] obesity[13] and/or metabolic syndrome.[14] These in turn may induce stress-related physiological changes related to the dysregulation of the hypothalamic-pituitary-adrenocortical axis and of the sympathetic nervous system; these enhance pro-inflammatory activity and may overactivate the renin-angiotensin-aldosterone system.[15] Chronic stress may also lead to unhealthy lifestyle.[16] Such physiological and behavioral changes may increase blood pressure (BP) and the risk of other cardiovascular diseases (e.g. ischemic heart diseases, stroke) or cardiac mortality,[17 18] already in young age.[2]

To our knowledge, only five studies investigated the association between death of a parent and BP and the findings have been inconsistent.[4 7 19-21] Luecken[7] and Anderson[20] reported that study participants who experienced parental death during childhood had higher systolic BP (SBP) and diastolic BP (DBP) than their unexposed counterparts. In contrast, Schooling[4] and Stein[21] found no association between parental death and BP or hypertension. In a study conducted later, Luecken et al. found that university students who lost their parent had lower 24-hour ambulatory BP compared to non-bereaved students.[19] Except for the studies conducted by Luecken et al., which assessed BP in childhood or in young adulthood,[7 19] all other studies were conducted in middle-aged individuals and assessed exposure retrospectively; [4 20 21] thus they could not eliminate selection bias, i.e., that severe hypertensive patients may die before middle-age. A further potential explanation for the discrepant findings in these earlier studies may be related to differences in their multivariate adjustments; some investigators did not adjust at all

For peer review only - http://bmjopen.bmj.com/site/about/guidelines.xhtml

for confounders,[7 19] some adjusted for a limited number of confounders,[4 21] while others adjusted for several factors that may be on the causal pathway between parental death and hypertension.[20]

None of these earlier studies investigated the importance of the type of the parent's death, analyses often conducted in bereavement research to attempt to separate the effect of stress from confounding or to investigate dose-response effects. The effect of exposure to parental death due to natural causes, in particular those due to cardiovascular diseases (CVD), on the risk of hypertension is likely to be confounded by genetic and environmental cardiovascular factors shared by family members, e.g., socioeconomic factors, lifestyle, mental health, cardiometabolic risk factors and morbidity; in contrast, the importance of familial confounding by cardiovascular risk factors is likely to be substantially more modest for unnatural deaths.[2 22] Furthermore, unnatural deaths have been suggested to be more strongly associated with stress and an increased risk of complicated grief than deaths due to natural causes.[6 16 23] Similarly, maternal death may have a more negative effect on BP than paternal death since in most cultural contexts mothers have stronger emotional bonds with their children, are more involved in their children's upbringing,[24] have a stronger impact on their children's health behavior and are likely to provide more emotional support for coping with grief than fathers.[25] Similarly, the first few years of life and adolescence could be particularly sensitive periods with respect to stress. Losing a parent in the first few years of life may be particularly detrimental[26] as warm relationships with caregivers are critical for the development of the brain architecture and the programming of stress reactivity,[7] while loss of a parent in adolescence, another period with an increased stress-sensitivity, may result in taking up adverse health behaviors that increase the CVD risk.[27]

High BP in youth tracks into adulthood and is an important predictor of later hypertension and CVD;[28 29] it is associated with increased risks of abnormal left ventricular mass,[30] metabolic

For peer review only - http://bmjopen.bmj.com/site/about/guidelines.xhtml

syndrome,[31] impaired cognitive ability,[32] renal damage[33] and cardiovascular and total mortality.[34 35] The prevalence of elevated BP among children and young adults has been increasing.[36 37] Though adverse childhood experiences (most often defined in terms of maltreatment and family dysfunction), are associated with an increased risk of hypertension in middle- and old age,[15 38 39] knowledge about their association with high BP in youth is more limited.[40-43]

We investigated the association between parental death during childhood − one of the most severe childhood adversities − and BP at the age of 18-20 years in a cohort of Swedish men. We also analyzed whether this association differs by the parent's gender, cause of death and the child's age at loss.

METHODS

Study population and design

This study was based on a cohort consisting of 49,321 men born in 1949-51 and who were conscripted for military service in Sweden in 1969-1970. The cohort includes 97-98% of all Swedish men of conscription age (≈18-20 years); the 2-3% that were exempted from participation had severe congenital disorders or intellectual disabilities.[44] At conscription, participants completed an extensive questionnaire and participated in a clinical examination performed by a team of physicians, psychiatrists and psychologists. The conscription data were linked to several nationwide registers through the unique personal number. We excluded participants with no information on exposure (n=314), missing BP (n=369) or higher DBP than SBP (n=14), resulting in 48,624 men being included in our analysis.

The study was approved by the Regional Ethics Board in Stockholm (reference numbers 2004-693/5, 2008/323-32 and 2010/604-32).

For peer review only - http://bmjopen.bmj.com/site/about/guidelines.xhtml

Measures

Exposure. Conscripts were linked to their parents using the Multigeneration Register. We obtained information on the date and the cause of the parent's death after 1952 from the Cause of Death Register; causes of death were recorded according to the International Classification of Diseases (ICD). We classified the cause of the parent's death as due to unnatural causes (ICD-6: 795 and 800-999; ICD-7: 795 and 800-999; ICD-8: 79599, 796 and 807-999), due to non-rheumatic CVD (ICD-6: 400-468; ICD-7: 400-468; ICD-8: 400-458) and due to other natural causes (the rest of the ICD codes). For the conscripts whose parents did not appear in the Cause of Death Register, we used two items from the questionnaire completed at conscription to determine their exposure status before 1952: "Are both of your parents alive" (yes/no), and "Whom have you mostly lived with" (with both parents/ mother/ father/ somebody else). If study participants responded "no" to the first question, the gender of the deceased parent was determined using the second question: (1) having lived with the mother was coded as paternal death, (2) having lived with the father was coded as maternal death, and (3) having lived with both parents or somebody else was coded as having no information on the deceased parent's gender.

Outcome. BP was measured at conscription according to a written protocol; the measurement was performed in a supine position, after a 5-10 minutes rest, with an appropriately sized cuff at the heart level.[44-46] Thus the measurement of BP in this study reflects the standard practice in primary care settings.[46] The measured SBP and the DBP values were rounded to the nearest even number, or to the nearest 5 or 10 mmHg. If the SBP was higher than 145 mmHg or if the DBP was lower than 50 mmHg or higher than 85 mmHg, another measurement was conducted the next day and the result of the second measurement was recorded; otherwise only one BP measurement was taken. Given that there is a linear association between BP in young age and the later risk of CVD,[47] that even BP values below 140/90 mmHg have a predictive value for

For peer review only - http://bmjopen.bmj.com/site/about/guidelines.xhtml

the development of later CVD, and since both SBP and DBP are associated with later CVD risk,[48 49] we considered the continuous SBP and DBP measures as the main outcome. In sensitivity analyses we also considered hypertension, defined as SBP ≥140 mmHg and/or DBP ≥90 mmHg.[50]

Covariates. The occupation of the head of the household (generally the father) from the National Population and Housing Censuses of 1960 was used as a measure of parental socioeconomic status (SES). Occupation was classified as: non-manual at middle or high level, non-manual employee at low level, skilled worker, unskilled worker, farmer, and other.[51] Men who during the psychological interview reported or were suspected to have a psychiatric disorder were referred to a psychiatrist for further evaluation. We retrieved information on depression using ICD-8 codes 296 and 300.4 and on anxiety using code 300.0, respectively. We calculated body mass index based on height and weight assessed at the clinical examination. Cardiorespiratory fitness was assessed using a cycle ergometer submaximal exercise test performed after a normal resting electrocardiogram. The work rate was increased until the participants were volitionally exhausted; the resulting maximal work capacity divided by weight was transformed to stanine scores (1-9).[45] The number of personal friends (0, 1-3, 3-5 or >5), the frequency of confidentially discussions with friends (never, sometimes or quite often), having a confidant to talk about personal problems (no one, or parent/siblings/teachers or supervisor or manager/friends/others), number of cigarettes per day (>10, 1-10, or 0), risky drinking behavior (defined as reporting any of the following: drinking > 250 g/week, having taken alcohol as an 'eye-opener' during a hangover, having been arrested for drunkenness, or having often been intoxicated)[52] and father's drinking habit (often, or sometimes/occasionally/never) were assessed by questionnaires. We calculated a cumulative index of childhood adversities by summing the number of the adverse childhood experiences reported by questionnaire from the following: financial situation of the family (bad/very bad

For peer review only - http://bmjopen.bmj.com/site/about/guidelines.xhtml

versus very good/good/average), paternal alcohol use (often versus never/occasionally/sometimes), parents' divorce (yes versus no), severe illness of parent (father/mother/both parents versus none), family member taking medicines for mental disorders (mother/father/both parents/others versus no one), experiencing physical punishment (often/sometimes/once in a while versus never), strict upbringing (very strict/quite strict versus medium/quite mild/very mild), and having multiple residences during childhood (more than three residences versus one/two/three residences). We considered participants whose answer was in the first group to have been exposed to the corresponding adverse experience. We chose these adverse childhood experiences based on previous literature − which often defined adverse childhood experiences in terms of abuse (emotional, physical and sexual), neglect or family dysfunction[15 53 54] − and the questionnaire completed at conscription. We calculated the cumulative index by summing the number of adverse events experienced, a common praxis in the literature[15 53 55] and based on evidence of a dose-response association between the number of adverse childhood experiences and the risk of CVD.[53 55]

Statistical analyses

We compared characteristics of exposed and unexposed men by chi-square tests in case of categorical variables and Wilcoxon tests in case of continuous variables with a skewed distribution. We used similar tests and logistic regression to study the link between covariates and our outcomes. We performed general linear regression to investigate the association between parental death and the continuous SBP and DBP measures. For each exposure category, we also estimated least square means of SBP and DBP and their 95% confidence intervals (CI). We performed analyses with any parental death during childhood and with exposure classified based on the cause of the parent's death, the gender of the deceased parent, and the age of the child when the parent died. We ran three models. Model 1 was unadjusted.

For peer review only - http://bmjopen.bmj.com/site/about/guidelines.xhtml

Model 2 was adjusted for childhood parental SES, a factor that is likely to be confounder of the investigated association. Model 3 was further adjusted for depression, anxiety, body mass index, cardiorespiratory fitness, number of friends, frequency of talking with friends confidentially, having a confidant, number of cigarettes smoked per day, and risky drinking behavior. We chose to run model 3 separately since we could not determine whether the variables in model 3 are confounders or mediators of the association under study; though their assessment after the exposure period may favor regarding them as mediators, several of these characteristics could have been present before the loss of a parent and may thus be confounders. To study whether father's drinking behavior confounded the association between death of a father and BP, we re-ran these analyses after adjusting for father's drinking behavior in addition to factors in model 3. To assure that the method of exposure assessment did not influence our results, we ran analyses after excluding the conscripts whose parent's death was identified only by questionnaire. We investigated effect modification by parental SES (classified as (1) non-manual employee versus (2) unskilled workers or skilled workers versus (3) farmer or other) and by our cumulative index of adverse childhood experiences (0, 1, 2 or ⩾3) by stratified analyses and formal tests of interaction. In further sensitivity analyses we run logistic regression models to investigate the association between death of a parent (any loss and exposure classified by the parent's cause of death, the deceased parent's gender and the child's age at loss) and hypertension. We deleted listwise in case of missing information on covariates.

We used SAS 9.4 for Windows for the analyses.

Patient and public involvement

Patients and/or the public were not involved in the design, conduct, reporting or dissemination plans of this research.

For peer review only - http://bmjopen.bmj.com/site/about/guidelines.xhtml

RESULTS

Of the 48624 men included in our study, 3504 (7.21%) experienced death of a parent before the age of 18. Characteristics of exposed and unexposed participants are shown in Table 1. The association between covariates and the risk of our outcomes is presented in Supplementary table 1.

The least square means of SBP and DBP were generally similar between the exposed and the unexposed groups, both when investigating any loss of a parent and when the exposure was classified according to the deceased parent's cause of death and gender, or the child's age at loss (Table 2). Men whose mother died due to unnatural causes had a slightly lower DBP than the unexposed in the unadjusted and the SES-adjusted, but not in the fully adjusted models (Table 3). The results did not change after excluding conscripts whose parent's death was defined through questionnaire-based information (Supplementary table 2). We found no strong evidence that parental SES or cumulative childhood adversity modified the association between parental death and BP (Supplementary table 3). Adjusting for father's drinking behavior in addition to factors in model 3 did not substantially change the association between death of a father and SBP or DBP (data not shown).

Death of a parent tended to be associated with a slightly increased odds of hypertension; the corresponding multivariate odds ratio (OR) (95% CI) was 1.10 (1.00-1.20). Losing a parent due to natural causes other than CVD was associated with hypertension. Losing a parent due to CVD also tended to be associated with hypertension. There was no association between parental death due to unnatural causes and the risk of hypertension. The point estimates corresponding to the association between death of a parent and hypertension did not differ substantially according to the parent's gender. Risks were slightly higher in case the child was 6-12 or 13-18 years at loss compared to when the loss occurred at earlier ages; these associations were confined only to losses due to natural causes (Table 4).

For peer review only - http://bmjopen.bmj.com/site/about/guidelines.xhtml

DISCUSSION

We found no strong evidence for an association between the death of a parent during childhood and SBP or DBP in early adulthood. The associations did not differ by the parent's cause of death, the gender of the deceased parent or the child's age at loss. Losing a parent was associated with a slightly increased risk of hypertension; the association was present only in case of losses due to natural causes.

Earlier studies investigating the association between parental death during childhood and elevated BP or hypertension have yielded mixed results. Two studies observed higher SBP and DBP among participants exposed to parental death during childhood than among their unexposed counterparts.[7 20] Two other studies found no relation between parental death and BP or hypertension,[4 21] whereas one study observed that students who experienced parental death had lower 24-hour ambulatory BP than non-bereaved students.[19] The reasons for these inconsistent findings are not clear but could be due to differences in study design, sample size, historical birth cohort, the age and the method of BP assessment and considerations about confounding by design or in multivariate models. Findings concerning the association between other types of childhood adversities – often defined as a cumulative index of events related to abuse, neglect or family dysfunction – and hypertension in middle- or old age are more consistent,[15 38 39] possibly due to the inclusion of a larger number of adverse life events and biases associated with the retrospective assessment of exposure. Our study extends knowledge in this field by focusing on BP assessed at a young age, using prospectively recorded information on parent's death from a high-quality nationwide register, rather than relying on retrospectively collected, questionnaire-based information on exposure, and analyzing a very large sample that allowed us to conduct sub-analyses to test hypotheses that may be supportive of a causal effect, if any.

For peer review only - http://bmjopen.bmj.com/site/about/guidelines.xhtml

Since an important challenge in studies regarding the association between bereavement and CVDs is related to the separation of the stress-related effect from confounding by genetic and environmental cardiovascular risk factors shared by family members, we performed analyses by the parents' cause of death classified as cardiovascular, other natural and unnatural deaths. Furthermore, to explore dose-response patterns and potential sensitive periods, we performed analyses according to the type of deceased parent or age at loss. We found no association between parental death during childhood and our continuous BP measures neither in case of overall exposure, nor when exposure was categorized by the parents' cause of death or gender or the child's age at loss. These results are indicative of a lack of a causal effect.

The finding that a modestly increased hypertension risk was observed only in case of parental deaths due to natural causes but not in case of parental deaths due to unnatural causes − which are often associated with more severe stress[6 16 23] − suggests that residual confounding by familial cardiovascular risk factors is a likely explanation for the association between parental death during childhood and hypertension. A family history of metabolic disorders and early CVD is a well-established risk factor of hypertension in children and youth.[56] An alternative explanation may be that severe stress increases the risk of hypertension in youth only among individuals with a genetic susceptibility to cardiometabolic disorders; children exposed to parental death due to natural causes are more likely to have such a susceptibility than children who lost a parent due to unnatural causes. A further though – in light of the fact that exposure in young age was not associated with the outcome – less likely explanation for the association between parental death due to natural causes and hypertension is that natural deaths may be proceeded by a long period of disease which may induce chronic stress for family members.[57]

The stronger association between parental death in adolescence and hypertension than in earlier periods of life – besides better statistical power – is also likely to be due to residual confounding,

For peer review only - http://bmjopen.bmj.com/site/about/guidelines.xhtml

given the higher proportion of parental deaths related to cardiometabolic conditions in the older age group.

A possible explanation for the limited evidence for an association between the death of a parent and BP in our study, in contrast to the large body of evidence documenting a link between psychological stress and hypertension in adulthood[58] and childhood adversities and hypertension in middle- or old age[15 38 39] may be related to the age of the BP assessment. Though children who lost a parent may experience chronic stress, the accumulation of further childhood (e.g. adverse socioeconomic circumstances, low social support, abuse or neglect, poor mental health) or adult adversities (e.g. low educational attainment, difficulties in attaching to a partner, difficulties on the labor marker) and the subsequent allostatic load may need to act for a longer period than what we studied to induce abnormal BP changes.[59] At the age of 18-20 years differences in BP may still be small across individuals.[43] Allostatic load may need to interact with the age-related vulnerability to increase BP later in life. This hypotheses is supported by findings of Su and associates who assessed BP on average 13 times between the age of 5 and 38 years, and found that mean SBP and DBP were similar in the first two decades of life among groups exposed and unexposed to adverse childhood experiences, but from the third decade of life differences among exposure groups in levels of BP became evident and with time increasingly important; the increase in mean BP was steeper with a higher number of adverse childhood experiences.[43]

Our findings need to be considered also in light of our study's limitations. First, since only one or two BP measurements were taken at conscription, we did not have the possibility to consider intra-individual variations in BP,[60] neither to reduce an eventual bias related to white-coat hypertension. Nevertheless, several measurements or ambulatory BP monitoring[19 61] may not be feasible in large epidemiological studies such as ours, as these measures may limit the sample size and may generate selection bias, e.g., children with parental death and/or hypertension may

For peer review only - http://bmjopen.bmj.com/site/about/guidelines.xhtml

be more or less likely to participate in such a study compared to others. The potential misclassification introduced by the single measurement in our study is likely to be non-differential and may eventually result in an underestimation of the true effect. Nevertheless, the BP measurement at conscription followed the standard measurement for screening for hypertension in primary care. Though one measurement of BP is not sufficient to confirm a clinical diagnosis of hypertension,[62] it has a screening value as the BP measure in this cohort has been reported to be an important predictor of later CVD and mortality.[46 63] Future studies with repeated measurements of BP ranging from childhood to later adulthood are needed to investigate the link between parental death and BP. Second, since the Conscription Cohort included only men, it is not clear whether the results can be generalized to women. Though women may be more sensitive to psychological stress with respect to hypertension than men,[64] earlier studies in this field investigating the association between adverse childhood experiences and youth BP have mixed results in term of gender differences.[43 65 66] Similarly, it is not clear to what extent our findings may be generalizable to current Swedish young adults or to populations with different risk factor profiles, support for bereaved children and healthcare.[67] Third, since information on most of our covariates was available only from conscription, we do not know whether they are confounders or mediators of the investigated associations. Though theoretical considerations and our measurement post- exposure favor regarding them as mediators, we cannot exclude the possibility that some of these measures are indicators of familial characteristics and may thus be proxies for confounding by factors that cluster in families.[17] Nevertheless, adjustment for factors in models 3 did not substantially affect our estimates; these covariates were generally weakly associated with hypertension. The finding that natural deaths but not unnatural deaths, which are less likely to be affected by familial cardiovascular risk, were associated with an increased hypertension risk and that associations were unchanged after adjusting for these suggests the presence of confounding by factors that

For peer review only - http://bmjopen.bmj.com/site/about/guidelines.xhtml

we did not consider; potential candidates include genetics, further socioeconomic factors, housing, diet and familial cardiometabolic disorders. Fourth, though our sample was very large, the power in some of our sub-analyses, e.g., when categorizing exposure according to the deceased parents' gender, cause of death and the child's age at loss and the risk of hypertension, may have been low to detect a modest effect at the conventional $p<0.05$ level.

We found no evidence that death of a parent was associated with continuous SBP and DBP measured at the age of 18-20. The fact that the association between loss of a parent and the modest risk of hypertension was observed in case of parental deaths due to natural causes but not in case of unnatural deaths, may be indicative of residual confounding by genetic and environmental factors shared by family members.

Contributors Conceptualization: HC, KDL, IJ. Data curation: HC, TH and KDL. Formal analysis: HC. Funding acquisition: KDL and HC. Methodology: HC, KDL, TH, IJ, MR, YF, LM and YL. Resources: TH and KDL. Supervision: KDL, TH, MR, and YF. Writing – original draft: HC. Writing – review and editing: HC, KDL, TH, IJ, MR, YF, YL and LM.

Funding The study was supported by the Swedish Council for Working Life and Social Research (grant number: 2015-00837), by Karolinska Institutet's Research Foundation (grant number: 2018-01924) and by the China Scholarship Council (grant number: 201700260296).

Competing interests None declared.

Ethics approval The study was approved by the Regional Ethics Board in Stockholm (reference numbers: 2004-693/5, 2008/323-32 and 2010/604-32).

Patient consent for publication Not required.

Data sharing statement Data may be obtained from a third party and are not publicly available.

For peer review only - http://bmjopen.bmj.com/site/about/guidelines.xhtml

REFERENCES

1. Breslau N, Wilcox HC, Storr CL*, et al.* Trauma exposure and posttraumatic stress disorder: a study of youths in urban America. *Journal of urban health : bulletin of the New York Academy of Medicine* 2004;81:530-44.

2. Li J, Vestergaard M, Cnattingius S*, et al.* Mortality after parental death in childhood: a nationwide cohort study from three Nordic countries. *PLoS medicine* 2014;11:e1001679.

3. Hjern A, Berg L, Arat A*, et al.* Children as next of kin in Sweden. BSA-rapport. Kalmar, 2017:28.

4. Schooling CM, Jiang C, Lam TH*, et al.* Parental death during childhood and adult cardiovascular risk in a developing country: the Guangzhou Biobank Cohort Study. *PloS one* 2011;6:e19675.

5. Sherr L, Croome N, Clucas C*, et al.* Differential effects of single and double parental death on child emotional functioning and daily life in South Africa. *Child welfare* 2014;93:149-72.

6. Dowdney L. Childhood bereavement following parental death. *Journal of child psychology and psychiatry, and allied disciplines* 2000;41:819-30.

7. Luecken LJ. Childhood attachment and loss experiences affect adult cardiovascular and cortisol function. *Psychosomatic medicine* 1998;60:765-72.

8. Berg L, Rostila M, Saarela J*, et al.* Parental death during childhood and subsequent school performance. *Pediatrics* 2014;133:682-9.

9. Geulayov G, Gunnell D, Holmen TL*, et al.* The association of parental fatal and non-fatal suicidal behaviour with offspring suicidal behaviour and depression: a systematic review and meta-analysis. *Psychological medicine* 2012;42:1567-80.

10. Berg L, Rostila M, Hjern A. Parental death during childhood and depression in young adults - a national cohort study. *Journal of child psychology and psychiatry, and allied disciplines* 2016;57:1092-8.

11. Hamdan S, Mazariegos D, Melhem NM*, et al.* Effect of parental bereavement on health risk behaviors in youth: a 3-year follow-up. *Archives of pediatrics & adolescent medicine* 2012;166:216-23.

For peer review only - http://bmjopen.bmj.com/site/about/guidelines.xhtml

12. Hamdan S, Melhem NM, Porta G*, et al.* Alcohol and substance abuse in parentally bereaved youth. *The Journal of clinical psychiatry* 2013;74:828-33.

13. Oliveira AJ, Rostila M, Saarela J*, et al.* The influence of bereavement on body mass index: results from a national Swedish survey. *PLoS One* 2014;9:e95201.

14. Alciati A, Gesuele F, Casazza G*, et al.* The relationship between childhood parental loss and metabolic syndrome in obese subjects. *Stress and health : journal of the International Society for the Investigation of Stress* 2013;29:5-13.

15. Obi IE, McPherson KC, Pollock JS. Childhood adversity and mechanistic links to hypertension risk in adulthood. *British journal of pharmacology* 2019;176:1932-50.

16. Stroebe M, Schut H, Stroebe W. Health outcomes of bereavement. *Lancet (London, England)* 2007;370:1960-73.

17. Chen H, Hemmingsson T, Forsell Y*, et al.* Death of a Parent during Childhood and the Risk of Ischemic Heart Disease and Stroke in Adult Men. *Psychosomatic medicine* 2020

18. Smith KR, Hanson HA, Norton MC*, et al.* Survival of offspring who experience early parental death: early life conditions and later-life mortality. *Social science & medicine (1982)* 2014;119:180-90.

19. Luecken LJ, Kraft A, Appelhans BM*, et al.* Emotional and cardiovascular sensitization to daily stress following childhood parental loss. *Dev Psychol* 2009;45:296-302.

20. Anderson EL, Caleyachetty R, Stafford M*, et al.* Prospective associations of psychosocial adversity in childhood with risk factors for cardiovascular disease in adulthood: the MRC National Survey of Health and Development. *International journal for equity in health* 2017;16:170.

21. Stein DJ, Scott K, Haro Abad JM*, et al.* Early childhood adversity and later hypertension: data from the World Mental Health Survey. *Annals of clinical psychiatry : official journal of the American Academy of Clinical Psychiatrists* 2010;22:19-28.

22. Rostila M, Berg L, Saarela J*, et al.* Experience of Sibling Death in Childhood and Risk of Death in Adulthood: A National Cohort Study From Sweden. *American journal of epidemiology* 2017;185:1247-54.

23. Brunner E. Stress mechanisms in coronary heart disease. 2002

For peer review only - http://bmjopen.bmj.com/site/about/guidelines.xhtml

24. Fuchs A, Mohler E, Reck C*, et al.* The Early Mother-to-Child Bond and Its Unique Prospective Contribution to Child Behavior Evaluated by Mothers and Teachers. *Psychopathology* 2016;49:211-16.

25. Kirwin KM, Hamrin V. Decreasing the risk of complicated bereavement and future psychiatric disorders in children. *Journal of child and adolescent psychiatric nursing : official publication of the Association of Child and Adolescent Psychiatric Nurses, Inc* 2005;18:62-78.

26. Koehn AJ, Kerns KA. Parent-child attachment: meta-analysis of associations with parenting behaviors in middle childhood and adolescence. *Attachment & human development* 2018;20:378-405.

27. Pabayo R, Molnar BE, Kawachi I. Witnessing a violent death and smoking, alcohol consumption, and marijuana use among adolescents. *Journal of urban health : bulletin of the New York Academy of Medicine* 2014;91:335-54.

28. Chen X, Wang Y. Tracking of blood pressure from childhood to adulthood: a systematic review and meta-regression analysis. *Circulation* 2008;117:3171-80.

29. Merai R, Siegel C, Rakotz M*, et al.* CDC grand rounds: A public health approach to detect and control hypertension. *Morbidity and Mortality Weekly Report* 2016;65:1261-64.

30. Falkner B, DeLoach S, Keith SW*, et al.* High risk blood pressure and obesity increase the risk for left ventricular hypertrophy in African-American adolescents. *The Journal of pediatrics* 2013;162:94-100.

31. Sun SS, Grave GD, Siervogel RM*, et al.* Systolic blood pressure in childhood predicts hypertension and metabolic syndrome later in life. *Pediatrics* 2007;119:237-46.

32. Rovio SP, Pahkala K, Nevalainen J*, et al.* Cardiovascular Risk Factors From Childhood and Midlife Cognitive Performance: The Young Finns Study. *Journal of the American College of Cardiology* 2017;69:2279-89.

33. Sundin PO, Udumyan R, Sjostrom P*, et al.* Predictors in adolescence of ESRD in middle-aged men. *American journal of kidney diseases : the official journal of the National Kidney Foundation* 2014;64:723-9.

For peer review only - http://bmjopen.bmj.com/site/about/guidelines.xhtml

34. Franklin BA, Chaddha A. Resting Heart Rate and Blood Pressure as Indices of Cardiovascular and Mortality Risk: IS LOWER INVARIABLY BETTER? *Journal of cardiopulmonary rehabilitation and prevention* 2018;38:353-57.

35. Lim SS, Vos T, Flaxman AD*, et al.* A comparative risk assessment of burden of disease and injury attributable to 67 risk factors and risk factor clusters in 21 regions, 1990-2010: a systematic analysis for the Global Burden of Disease Study 2010. *Lancet (London, England)* 2012;380:2224-60.

36. Kit BK, Kuklina E, Carroll MD*, et al.* Prevalence of and trends in dyslipidemia and blood pressure among US children and adolescents, 1999-2012. *JAMA pediatrics* 2015;169:272-9.

37. Noubiap JJ, Essouma M, Bigna JJ*, et al.* Prevalence of elevated blood pressure in children and adolescents in Africa: a systematic review and meta-analysis. *The Lancet Public health* 2017;2:e375-e86.

38. Springer KW, Sheridan J, Kuo D*, et al.* Long-term physical and mental health consequences of childhood physical abuse: results from a large population-based sample of men and women. *Child abuse & neglect* 2007;31:517-30.

39. Parrish C, Surkan PJ, Martins SS*, et al.* Childhood adversity and adult onset of hypertension and heart disease in Sao Paulo, Brazil. *Preventing chronic disease* 2013;10:E205.

40. Gooding HC, Milliren CE, Austin SB*, et al.* Child Abuse, Resting Blood Pressure, and Blood Pressure Reactivity to Psychosocial Stress. *Journal of pediatric psychology* 2016;41:5-14.

41. Doom JR, Mason SM, Suglia SF*, et al.* Pathways between childhood/adolescent adversity, adolescent socioeconomic status, and long-term cardiovascular disease risk in young adulthood. *Social science & medicine (1982)* 2017;188:166-75.

42. Su S, Wang X, Kapuku GK*, et al.* Adverse childhood experiences are associated with detrimental hemodynamics and elevated circulating endothelin-1 in adolescents and young adults. *Hypertension (Dallas, Tex : 1979)* 2014;64:201-7.

43. Su S, Wang X, Pollock JS*, et al.* Adverse childhood experiences and blood pressure trajectories from childhood to young adulthood: the Georgia stress and Heart study. *Circulation* 2015;131:1674-81.

For peer review only - http://bmjopen.bmj.com/site/about/guidelines.xhtml

44. Falkstedt D, Hemmingsson T, Rasmussen F*, et al.* Body mass index in late adolescence and its association with coronary heart disease and stroke in middle age among Swedish men. *International journal of obesity (2005)* 2007;31:777-83.

45. Lindgren M, Robertson J, Adiels M*, et al.* Resting heart rate in late adolescence and long term risk of cardiovascular disease in Swedish men. *International journal of cardiology* 2018;259:109-15.

46. Sundstrom J, Neovius M, Tynelius P*, et al.* Association of blood pressure in late adolescence with subsequent mortality: cohort study of Swedish male conscripts. *BMJ (Clinical research ed)* 2011;342:d643.

47. Britton KA, Gaziano JM, Djousse L. Normal systolic blood pressure and risk of heart failure in US male physicians. *European journal of heart failure* 2009;11:1129-34.

48. Muntner P, Shimbo D, Carey RM*, et al.* Measurement of Blood Pressure in Humans: A Scientific Statement From the American Heart Association. *Hypertension (Dallas, Tex : 1979)* 2019;73:e35-e66.

49. Flint AC, Conell C, Ren X*, et al.* Effect of Systolic and Diastolic Blood Pressure on Cardiovascular Outcomes. *The New England journal of medicine* 2019;381:243-51.

50. Whelton Pk Fau - Carey RM, Carey Rm Fau - Aronow WS, Aronow Ws Fau - Casey DE, Jr.*, et al.* 2017 ACC/AHA/AAPA/ABC/ACPM/AGS/APhA/ASH/ASPC/NMA/PCNA Guideline for the Prevention, Detection, Evaluation, and Management of High Blood Pressure in Adults: A Report of the American College of Cardiology/American Heart Association Task Force on Clinical Practice Guidelines.

51. Falkstedt D, Lundberg I, Hemmingsson T. Childhood socio-economic position and risk of coronary heart disease in middle age: a study of 49,321 male conscripts. *European journal of public health* 2011;21:713-8.

52. Larsson D, Hemmingsson T, Allebeck P*, et al.* Self-rated health and mortality among young men: what is the relation and how may it be explained? *Scandinavian journal of public health* 2002;30:259-66.

For peer review only - http://bmjopen.bmj.com/site/about/guidelines.xhtml

53. Suglia SF, Koenen KC, Boynton-Jarrett R*, et al.* Childhood and Adolescent Adversity and Cardiometabolic Outcomes: A Scientific Statement From the American Heart Association. *Circulation* 2018;137:e15-e28.

54. Hughes K, Bellis MA, Hardcastle KA*, et al.* The effect of multiple adverse childhood experiences on health: a systematic review and meta-analysis. *The Lancet Public health* 2017;2:e356-e66.

55. Felitti VJ. The Relation Between Adverse Childhood Experiences and Adult Health: Turning Gold into Lead. *The Permanente journal* 2002;6:44-47.

56. Kawabe H, Azegami T, Takeda A*, et al.* Features of and preventive measures against hypertension in the young.

57. Chen R, Fall K, Czene K*, et al.* Impact of parental cancer on IQ, stress resilience, and physical fitness in young men. *Clinical epidemiology* 2018;10:593-604.

58. Liu MY, Li N, Li WA*, et al.* Association between psychosocial stress and hypertension: a systematic review and meta-analysis.

59. Kuh D, Ben-Shlomo Y. A life course approach to chronic disease epidemiology. 2 ed. Oxford: Oxford University Press 2004.

60. Islam MS. Hypertension: From Basic Research to Clinical Practice. *Advances in experimental medicine and biology* 2017;956:1-2.

61. Buckley T, Mihailidou AS, Bartrop R*, et al.* Haemodynamic changes during early bereavement: potential contribution to increased cardiovascular risk. *Heart, lung & circulation* 2011;20:91-8.

62. Flynn JT, Kaelber DC, Baker-Smith CM*, et al.* Clinical Practice Guideline for Screening and Management of High Blood Pressure in Children and Adolescents. *Pediatrics* 2017;140

63. Falkstedt D, Koupil I, Hemmingsson T. Blood pressure in late adolescence and early incidence of coronary heart disease and stroke in the Swedish 1969 conscription cohort. *Journal of hypertension* 2008;26:1313-20.

64. Di Pilla M, Bruno RM, Taddei S*, et al.* Gender differences in the relationships between psychosocial factors and hypertension. *Maturitas* 2016;93:58-64.

65. Dietz LJ, Pham S, Melhem N*, et al.* Blood pressure recovery to social stress in parentally bereaved and non-bereaved youths. *Journal of psychosomatic research* 2018;113:58-65.

For peer review only - http://bmjopen.bmj.com/site/about/guidelines.xhtml

66. Luecken LJ, Roubinov DS. Hostile behavior links negative childhood family relationships to heart rate reactivity and recovery in young adulthood. *International journal of psychophysiology : official journal of the International Organization of Psychophysiology* 2012;84:172-9.

67. Rostila M. Commentary: Childhood parental loss and adulthood health: discussing the role of parental cause of death, child's age at death and historical context. *Social science & medicine (1982)* 2015;131:190-2.

For peer review only

For peer review only - http://bmjopen.bmj.com/site/about/guidelines.xhtml

Table1. Characteristics of study participants according to death of a parent (N=48624)

Variables	Total, n (%)	Death of a parent Yes (n=3504)	Death of a parent No (n=45120)	*P*-value*
Categorical variables, n (%)				
Parental socioeconomic status				<0.01
Middle or high level non-manual employee	10716 (22.0)	689 (19.7)	10027 (22.2)	
Low level non-manual employee	4942 (10.2)	300 (8.6)	4642 (10.3)	
Skilled worker	10403 (21.4)	510 (14.6)	9893 (21.9)	
Unskilled worker	16094 (33.1)	1121 (32.0)	14973 (33.2)	
Farmer	5373 (11.0)	399 (11.4)	4974 (11.0)	
Other	1096 (2.3)	485 (13.8)	611 (1.4)	
Depression				<0.01
No	47892 (98.5)	3421 (97.6)	44471 (98.6)	
Yes	732 (1.5)	83 (2.4)	649 (1.4)	
Anxiety				0.72
No	48469 (99.7)	3494 (99.7)	44975 (99.7)	
Yes	155 (0.3)	10 (0.3)	145 (0.3)	
Cardiorespiratory fitness (stanine scores)				<0.01
1	34 (0.1)	2 (0.1)	32 (0.1)	
2	207 (0.4)	15 (0.4)	192 (0.4)	
3	2451 (5.0)	207 (5.9)	2244 (5.0)	
4	7020 (14.4)	578 (16.5)	6442 (14.3)	
5	11447 (23.5)	857 (24.4)	10590 (23.5)	

For peer review only - http://bmjopen.bmj.com/site/about/guidelines.xhtml

6	9215 (19.0)	658 (18.8)	8557 (19.0)	
7	5713 (11.8)	401 (11.4)	5312 (11.8)	
8	4608 (9.5)	305 (8.7)	4303 (9.5)	
9	7867 (16.2)	480 (13.7)	7387 (16.4)	
Missing	62 (0.1)	1 (0.1)	61 (0.1)	
Number of personal friends				0.72
0	389 (0.8)	29 (0.8)	360 (0.8)	
1-3	3260 (6.7)	240 (6.8)	3020 (6.7)	
3-5	15195 (31.3)	1114 (31.8)	14081 (31.2)	
>5	29006 (59.6)	2048 (58.4)	26958 (59.7)	
Missing	774 (1.6)	73 (2.1)	701 (1.6)	
Confidential discussions with friends				<0.01
Never	2011 (4.2)	178 (5.1)	1833 (4.1)	
Sometime	28655 (58.9)	2061 (58.8)	26594 (58.9)	
Quite often	17072 (35.1)	1186 (33.8)	15886 (35.2)	
Missing	886 (1.8)	79 (2.3)	807 (1.8)	
Has a confidant				<0.01
No	7865 (16.2)	633 (18.1)	7232 (16.0)	
Yes	39741 (81.7)	2772 (79.1)	36969 (81.9)	
Missing	1018 (2.1)	99 (2.8)	919 (2.0)	
Number of cigarettes smoked per day				
0	19903 (40.9)	1216 (34.7)	18687 (41.4)	<0.01
1-10	15310 (31.5)	1150 (32.8)	14160 (31.4)	
>10	12731 (26.2)	1067 (30.5)	11664 (25.9)	
Missing	680 (1.4)	71 (2.0)	609 (1.3)	

For peer review only - http://bmjopen.bmj.com/site/about/guidelines.xhtml

For peer review only

Risky drinking behavior				<0.01
No	42323 (87.0)	2923 (83.4)	39400 (87.3)	
Yes	6267 (12.9)	579 (16.5)	5688 (12.6)	
Missing	34 (0.1)	2 (0.1)	32 (0.1)	
Father's drinking habits				<0.01
Never, occasionally or sometimes	45580 (93.8)	3048 (87.0)	42532 (94.3)	
Often	1954 (4.0)	175 (5.0)	1779 (3.9)	
Missing	1090 (2.2)	281 (8.0)	809 (1.8)	
Cumulative adverse childhood experiences				<0.01
0	9617 (19.8)	482 (13.8)	9135 (20.2)	
1	18477 (38.0)	1043 (29.8)	17434 (38.6)	
2	12173 (25.0)	1061 (30.3)	11112 (24.6)	
⩾3	8357 (17.2)	918 (26.2)	7439 (16.5)	
Continuous variable, median				
Body mass index, kg/m^2	20.6	20.6	20.7	0.71

*Men with no missing data on the corresponding variables were included in these analyses.

For peer review only

Table 2. Least square means and 95% confidence intervals for blood pressure according to exposure to death of a parent during childhood (N=48624)

Type of exposure	Model 1*		Model 2†		Model 3‡	
	LS Mean (95% CI)	*P*-value	LS Mean (95% CI)	*P*-value	LS Mean (95% CI)	*P*-value
**Systolic blood pressure, mmHg**						
Unexposed	126.09 (125.99-126.20)	-	126.13 (125.97-126.29)	-	128.02 (126.86-129.18)	-
Any loss	126.34 (125.95-126.72)	0.24	126.35 (125.96-126.74)	0.30	128.25 (127.04-129.46)	0.26
Cause of death of the parent§						
Unnatural death	125.49 (124.58-126.40)	0.20	125.56 (124.65-126.47)	0.23	127.57 (126.10-129.03)	0.59
Cardiovascular death	126.94 (126.01-127.87)	0.08	126.99 (126.06-127.92)	0.07	128.40 (126.92-129.88)	0.22
Other natural cause	126.62 (126.05-127.19)	0.08	126.59 (126.02-127.16)	0.12	128.31 (127.02-129.59)	0.10
Gender of deceased parent§						
Mother	126.36 (125.59-127.14)	0.50	126.26 (125.47-127.04)	0.74	128.14 (126.74-129.53)	0.51
Father	126.50 (126.00-126.99)	0.12	126.55 (126.06-127.05)	0.10	128.30 (127.04-129.55)	0.11
Child's age at loss						
≤5 years	125.93 (125.24-126.62)	0.65	125.96 (125.26-126.65)	0.60	127.87 (126.54-129.20)	0.63

For peer review only - http://bmjopen.bmj.com/site/about/guidelines.xhtml

6-12 years	126.30 (125.57-127.04)	0.58	126.30 (125.56-127.03)	0.69	128.15 (126.79-129.51)	0.77
13-18 years	126.67 (126.06-127.28)	0.07	126.69 (126.08-127.31)	0.08	128.64 (127.34-129.94)	0.05
**Diastolic blood pressure, mmHg**						
Unexposed	72.90 (72.82-72.99)	-	72.94 (72.81-73.07)		73.99 (73.06-74.93)	-
Any loss	72.88 (72.57-73.19)	0.87	72.86 (72.55-73.17)	0.63	73.86 (72.89-74.84)	0.45
Cause of death of the parent§						
Unnatural death	72.16 (71.43-72.88)	0.05	72.10 (71.37-72.82)	0.02	73.20 (72.02-74.39)	0.06
Cardiovascular death	73.42 (72.68-74.16)	0.18	73.40 (72.66-74.14)	0.24	74.14 (72.94-75.33)	0.60
Other natural cause	73.13 (72.67-73.58)	0.34	73.12 (72.67-73.58)	0.46	74.14 (73.10-75.18)	0.40
Gender of deceased parent§						
Mother	72.36 (71.74-72.97)	0.08	72.39 (71.76-73.01)	0.09	73.43 (72.31-74.56)	0.16
Father	73.16 (72.77-73.55)	0.20	73.11 (72.72-73.51)	0.38	74.00 (72.99-75.01)	0.58
Child's age at loss						
≤5 years	72.62 (72.07-73.17)	0.32	72.57 (72.02-73.12)	0.20	73.49 (72.42-74.57)	0.08
6-12 years	73.04 (72.46-73.62)	0.66	72.99 (72.41-73.57)	0.88	73.98 (72.88-75.08)	0.93
13-18 years	72.97 (72.49-73.45)	0.80	72.99 (72.50-73.49)	0.84	74.09 (73.04-75.14)	0.74

For peer review only - http://bmjopen.bmj.com/site/about/guidelines.xhtml

LS Mean=least square mean; CI=confidence interval.

*Model 1 was unadjusted.

†Model 2 was adjusted for parental socioeconomic status.

‡Model 3 was adjusted for parental socioeconomic status, depression, anxiety, body-mass index, cardiorespiratory fitness, number of friends, frequency of talking with friends confidentially, having a confidant, number of cigarettes smoked per day, and risky drinking behavior.

§Only men with no missing data on this type of exposure were included.

For peer review only

For peer review only - http://bmjopen.bmj.com/site/about/guidelines.xhtml

Table 3. Least square means and 95% confidence intervals for systolic blood pressure and diastolic blood pressure, by gender of the deceased parent during childhood and further classified according to the cause of death and the age at loss (N=48624)

Type of exposure	Model 1*		Model 2†		Model 3‡	
	LS Mean (95% CI)	*P*-value	LS Mean (95% CI)	*P*-value	LS Mean (95% CI)	*P*-value
**Systolic blood pressure, mmHg**						
Unexposed	126.09 (125.99-126.20)	-	126.12 (125.96-126.28)	-	127.81 (126.65-128.98)	-
Death of a mother§						
Cause of death						
Unnatural death	125.35 (123.05-127.66)	0.53	125.57 (123.27-127.87)	0.64	127.43 (124.84-130.02)	0.75
Cardiovascular death	126.42 (123.84-129.00)	0.81	126.30 (123.73-128.87)	0.89	127.87 (125.00-130.73)	0.97
Other natural death	126.62 (125.72-127.51)	0.25	126.47 (125.57-127.37)	0.44	128.27 (126.81-129.73)	0.32
Child's age at loss						
≤5 years	124.99 (123.15-126.84)	0.24	124.75 (122.91-126.59)	0.14	126.55 (124.36-128.74)	0.16
6-12 years	126.29 (125.01-127.56)	0.77	126.16 (124.89-127.44)	0.95	127.79 (126.07-129.51)	0.91
13-18 years	126.95 (125.80-128.10)	0.14	126.91 (125.76-128.07)	0.18	129.00 (127.37-130.63)	0.05
Death of a father§						

For peer review only - http://bmjopen.bmj.com/site/about/guidelines.xhtml

Cause of death						
Unnatural death	125.51 (124.52-126.51)	0.25	125.56 (124.57-126.55)	0.27	127.59 (126.07-129.11)	0.66
Cardiovascular death	127.02 (126.02-128.02)	0.07	127.10 (126.10-128.09)	0.06	128.47 (126.95-129.99)	0.20
Other natural death	126.62 (125.88-127.37)	0.17	126.68 (125.93-127.42)	0.15	128.33 (126.96-129.70)	0.18
Child's age at loss						
≤5 years	126.60 (125.58-127.63)	0.33	126.74 (125.70-127.77)	0.26	128.55 (127.01-130.09)	0.21
6-12 years	126.31 (125.41-127.20)	0.64	126.38 (125.48-127.28)	0.59	128.13 (126.66-129.59)	0.59
13-18 years	126.56 (125.85-127.28)	0.20	126.58 (125.86-127.30)	0.22	128.28 (126.92-129.63)	0.27
**Diastolic blood pressure, mmHg**						
Unexposed	72.90 (72.82-72.99)	-	72.93 (72.80-73.06)	-	73.92 (72.98-74.86)	-
Death of a mother§						
Cause of death						
Unnatural death	70.68 (68.84-72.51)	0.02	70.67 (68.84-72.51)	0.02	72.28 (70.18-74.37)	0.09
Cardiovascular death	72.09 (70.04-74.14)	0.44	72.13 (70.07-74.18)	0.44	72.61 (70.29-74.92)	0.22
Other natural death	72.69 (71.98-73.40)	0.55	72.73 (72.01-73.45)	0.58	73.81 (72.63-74.99)	0.77
Child's age at loss						

For peer review only - http://bmjopen.bmj.com/site/about/guidelines.xhtml

≤5 years	71.83 (70.37-73.30)	0.15	71.86 (70.40-73.33)	0.16	74.35 (73.12-75.58)	0.08
6-12 years	72.16 (71.14-73.17)	0.15	72.20 (71.18-73.22)	0.16	74.35 (73.25-75.46)	0.17
13-18 years	72.72 (71.80-73.63)	0.69	72.74 (71.82-73.66)	0.69	73.37 (72.14-74.60)	0.81
Death of a father§						
Cause of death						
Unnatural death	72.43 (71.64-73.22)	0.24	72.36 (71.57-73.15)	0.17	74.00 (72.69-75.31)	0.18
Cardiovascular death	73.62 (72.82-74.41)	0.08	73.59 (72.79-74.38)	0.11	72.52 (70.75-74.29)	0.30
Other natural death	73.44 (72.85-74.03)	0.08	73.39 (72.80-73.99)	0.14	73.16 (71.77-74.55)	0.17
Child's age at loss						
≤5 years	72.96 (72.14-73.78)	0.90	72.84 (72.01-73.67)	0.84	73.76 (72.52-75.01)	0.77
6-12 years	73.47 (72.76-74.19)	0.12	73.39 (72.67-74.11)	0.22	74.25 (73.07-75.43)	0.34
13-18 years	73.07 (72.50-73.64)	0.58	73.07 (72.50-73.65)	0.62	73.96 (72.87-75.06)	0.79

LS Mean=least square mean; CI=confidence interval.

*Model 1 was unadjusted.

†Model 2 was adjusted for parental socioeconomic status.

For peer review only

‡Model 3 was adjusted for parental socioeconomic status, depression, anxiety, body-mass index, cardiorespiratory fitness, number of friends, frequency of talking with friends confidentially, having a confidant, number of cigarettes smoked per day, and risky drinking behavior.

§Only men without missing data on type of exposure were include

For peer review only

For peer review only - http://bmjopen.bmj.com/site/about/guidelines.xhtml

Table 4. Odds ratios for high blood pressure at conscription according to exposure to death of a parent during childhood (N=48624)

Type of exposure	Events/N	Model 1*	Model 2†	Model 3‡
		OR (95% CI)	OR (95% CI)	OR (95% CI)
Unexposed	9166/45120	1.00	1.00	1.00
Any loss	776/3504	1.12 (1.03-1.21)	1.11 (1.02-1.21)	1.10 (1.00-1.20)
Cause of death of the parent§				
Unnatural death	119/634	0.91 (0.74-1.11)	0.91 (0.74-1.11)	0.93 (0.75-1.15)
Cardiovascular death	142/606	1.20 (0.99-1.45)	1.20 (0.99-1.45)	1.15 (0.95-1.40)
Other natural cause	383/1608	1.23 (1.09-1.38)	1.21 (1.08-1.37)	1.20 (1.06-1.35)
Child's age at loss				
≤5 years	218/1101	0.97 (0.83-1.13)	0.96 (0.82-1.12)	0.94 (0.81-1.11)
6-12 years	226/979	1.18 (1.01-1.37)	1.16 (1.00-1.36)	1.15 (0.98-1.35)
13-18 years	332/1424	1.19 (1.05-1.35)	1.19 (1.05-1.35)	1.18 (1.03-1.34)
Gender of deceased parent§				
Mother	196/876	1.13 (0.96-1.33)	1.11 (0.95-1.30)	1.12 (0.94-1.32)
Cause of mother's death				
Unnatural death	21/99	1.06 (0.65-1.71)	1.08 (0.67-1.76)	1.06 (0.64-1.77)
Cardiovascular death	14/79	0.84 (0.47-1.51)	0.83 (0.46-1.48)	0.88 (0.49-1.60)
Other natural death	155/660	1.20 (1.00-1.44)	1.18 (0.98-1.41)	1.18 (0.98-1.42)
Child's age at mother's death				
≤5 years	25/155	0.75 (0.49-1.16)	0.73 (0.47-1.12)	0.65 (0.41-1.04)
6-12 years	81/323	1.31 (1.02-1.69)	1.29 (1.00-1.66)	1.27 (0.97-1.65)
13-18 years	90/398	1.15 (0.91-1.45)	1.14 (0.90-1.44)	1.19 (0.93-1.52)
Father	493/2180	1.15 (1.03-1.27)	1.15 (1.03-1.28)	1.13 (1.01-1.26)

For peer review only

For peer review only - http://bmjopen.bmj.com/site/about/guidelines.xhtml

Cause of father's death				
Unnatural death	98/535	0.88 (0.71-1.10)	0.88 (0.70-1.10)	0.91 (0.72-1.14)
Cardiovascular death	128/527	1.26 (1.03-1.54)	1.26 (1.03-1.54)	1.19 (0.97-1.47)
Other natural death	228/948	1.24 (1.07-1.44)	1.24 (1.06-1.45)	1.21 (1.03-1.42)
Child's age at father's death				
≤5 years	106/498	1.06 (0.85-1.32)	1.06 (0.85-1.33)	1.06 (0.84-1.33)
6-12 years	145/656	1.11 (0.92-1.34)	1.11 (0.92-1.34)	1.10 (0.90-1.34)
13-18 years	242/1026	1.21 (1.05-1.40)	1.21 (1.05-1.40)	1.17 (1.01-1.37)
Child's age at loss§				
≤5 years				
Cause of parent's death				
Unnatural death	17/126	0.61 (0.37-1.02)	0.61 (0.36-1.02)	0.61 (0.36-1.05)
Cardiovascular death	12/61	0.96 (0.51-1.81)	0.97 (0.52-1.84)	1.01 (0.53-1.93)
Other natural death	57/258	1.11 (0.83-1.49)	1.09 (0.81-1.47)	1.04 (0.76-1.41)
6-12 years				
Cause of parent's death				
Unnatural death	41/229	0.86 (0.61-1.20)	0.84 (0.60-1.19)	0.85 (0.59-1.21)
Cardiovascular death	46/185	1.30 (0.93-1.81)	1.29 (0.92-1.81)	1.24 (0.88-1.77)
Other natural death	139/565	1.28 (1.06-1.55)	1.26 (1.04-1.53)	1.24 (1.01-1.52)
13-18 years				
Cause of parent's death				
Unnatural death	61/279	1.10 (0.83-1.46)	1.11 (0.84-1.48)	1.15 (0.86-1.54)
Cardiovascular death	84/360	1.19 (0.93-1.53)	1.19 (0.93-1.52)	1.12 (0.87-1.45)
Other natural death	187/785	1.23 (1.04-1.45)	1.22 (1.03-1.44)	1.21 (1.02-1.44)

OR=odds ratio, CI=confidence interval.

For peer review only - http://bmjopen.bmj.com/site/about/guidelines.xhtml

*Model 1 was unadjusted.

†Model 2 was adjusted for parental socioeconomic status.

‡Model 3 was adjusted for parental socioeconomic status, depression, anxiety, body-mass index, cardiorespiratory fitness, number of friends, frequency of talking with friends confidentially, having a confidant, number of cigarettes smoked per day, and risky drinking behavior.

§Only men with no missing data on this type of exposure were included.

For peer review only

For peer review only - http://bmjopen.bmj.com/site/about/guidelines.xhtml

Supplementary table 1. Association between the covariates and blood pressure (N=48624)

Covariates	Systolic blood pressure		Diastolic blood pressure		Hypertension
	LS Mean (95% CI)*	*P*-value*	LS Mean (95% CI)*	*P*-value*	OR (95% CI)*
Parental socioeconomic status					
Middle or high level non-manual employee	124.63 (124.41-124.85)	-	72.92 (72.75-73.10)	-	1.00
Low level non-manual employee	125.26 (124.94-125.59)	<0.01	72.93 (72.67-73.19)	0.99	1.09 (1.00-1.19)
Skilled worker	126.68 (126.46-126.91)	<0.01	72.92 (72.74-73.10)	0.98	1.25 (1.17-1.34)
Unskilled worker	126.48 (126.30-126.66)	<0.01	72.98 (72.83-73.12)	0.64	1.25 (1.17-1.33)
Farmer	127.59 (127.28-127.90)	<0.01	72.48 (72.24-72.73)	<0.01	1.51 (1.39-1.63)
Other	126.31 (125.62-127.00)	<0.01	73.34 (72.79-73.90)	0.16	1.28 (1.10-1.49)
Depression					
No	126.10 (126.00-126.21)	-	72.90 (72.81-72.98)	-	1.00
Yes	126.76 (125.91-127.61)	0.13	73.31 (72.63-73.98)	0.24	1.20 (1.01-1.42)
Anxiety					
No	126.10 (126.00-126.21)	-	72.90 (72.82-72.98)	-	1.00
Yes	128.90 (127.05-130.74)	<0.01	73.76 (72.30-75.23)	0.25	1.18 (0.81-1.71)

For peer review only

Cardiorespiratory fitness (stanine scores)					
1	124.62 (120.68-128.56)	0.38	74.41 (71.28-77.54)	0.29	1.43 (0.67-3.08)
2	124.57 (122.97-126.17)	0.03	72.43 (71.16-73.69)	0.64	0.89 (0.63-1.28)
3	125.48 (125.02-125.95)	<0.01	72.90 (72.53-73.27)	0.43	1.00 (0.89-1.12)
4	125.92 (125.65-126.19)	0.01	73.08 (72.87-73.30)	0.02	1.02 (0.94-1.10)
5	126.06 (125.84-126.27)	0.05	73.01 (72.84-73.18)	0.04	1.02 (0.95-1.09)
6	126.14 (125.90-126.38)	0.16	72.82 (72.63-73.01)	0.54	1.04 (0.97-1.12)
7	126.43 (126.13-126.73)	0.85	73.00 (72.76-73.24)	0.10	1.05 (0.97-1.15)
8	126.01 (125.68-126.35)	0.08	72.69 (72.42-72.96)	0.82	1.02 (0.93-1.12)
9	126.39 (126.13-126.65)	-	72.73 (72.53-72.94)	-	1.00
Number of personal friends					
0	124.97 (123.81-126.14)	0.07	72.61 (71.68-73.53)	0.56	0.78 (0.60-1.02)
1-3	126.25 (125.85-126.66)	0.37	72.75 (72.43-73.07)	0.44	1.00 (0.92-1.10)
3-5	126.26 (126.08-126.45)	0.09	72.89 (72.74-73.04)	0.94	1.02 (0.97-1.07)
>5	126.06 (125.93-126.20)	-	72.88 (72.77-72.99)	-	1.00
Confidential discussions with friends					

For peer review only

Never	126.45 (125.93-126.96)	<0.01	73.26 (72.85-73.66)	0.07	1.18 (1.06-1.32)
Sometime	126.36 (126.23-126.50)	<0.01	72.85 (72.74-72.95)	0.90	1.09 (1.04-1.14)
Quite often	125.68 (125.51-125.86)	-	72.86 (72.72-73.00)	-	1.00
Has a confidant					
No	126.05 (125.79-126.30)	0.46	72.99 (72.78-73.19)	0.24	0.97 (0.91-1.03)
Yes	126.15 (126.04-126.27)	-	72.85 (72.76-72.94)	-	1.00
Number of cigarettes smoked per day					
0	127.26 (127.10-127.42)	-	73.60 (73.47-73.73)	-	1.00
1-10	125.55 (125.37-125.74)	<0.01	72.46 (72.32-72.61)	<0.01	0.73 (0.69-0.77)
>10	125.05 (124.85-125.25)	<0.01	72.22 (72.06-72.38)	<0.01	0.66 (0.63-0.70)
Risky drinking behavior					
No	126.17 (126.06-126.28)	-	72.90 (72.81-72.98)	-	1.00
Yes	125.71 (125.42-126.00)	<0.01	72.96 (72.73-73.19)	0.63	0.91 (0.85-0.98)
Body mass index, kg/m^2†	-	-	-	-	1.13 (1.12-1.14)

LS Mean=least square mean; CI=confidence interval; OR=odds ratio.

*Unadjusted model.

For peer review only

†In the linear regression on body mass index and systolic blood pressure, the estimated parameter was 0.87 and *p*-value was <0.01; in the linear regression on body mass index and diastolic blood pressure, the estimated parameter was 0.40 and *p*-value was <0.01.

For peer review only

Supplementary table 2. Associations between death of a parent during childhood and least square means for systolic and diastolic blood pressure at conscription after excluding exposed study participants whose exposure status was defined using questionnaire data (N= 47968)

Type of exposure	Model 1*		Model 2†		Model 3‡	
	LS Mean (95% CI)	*P*-value	LS Mean (95% CI)	*P*-value	LS Mean (95% CI)	*P*-value
**Systolic blood pressure**						
Unexposed	126.09 (125.99-126.20)	-	126.12 (125.96-126.28)	-	127.81 (126.65-128.98)	-
Any loss	126.44 (126.01-126.87)	0.13	126.45 (126.02-126.88)	0.15	128.16 (126.93-129.39)	0.13
Cause of death of the parent						
Unnatural death	125.49 (124.58-126.40)	0.20	125.56 (124.65-126.47)	0.23	127.57 (126.10-129.03)	0.59
Cardiovascular death	126.94 (126.01-127.87)	0.08	126.99 (126.06-127.92)	0.07	128.40 (126.92-129.88)	0.22
Other natural cause	126.62 (126.05-127.19)	0.08	126.59 (126.02-127.16)	0.12	128.31 (127.02-129.59)	0.10
Gender of deceased parent						
Mother	126.45 (125.66-127.24)	0.38	126.35 (125.55-127.14)	0.57	128.13 (126.73-129.54)	0.43
Father	126.43 (125.92-126.94)	0.20	126.49 (125.98-127.00)	0.17	128.17 (126.91-129.43)	0.19
Child's age at loss						
≤5 years	125.98 (124.90-127.07)	0.84	126.04 (124.95-127.13)	0.87	127.75 (126.17-129.33)	0.89

For peer review only

6-12 years	126.30 (125.57-127.03)	0.58	126.30 (125.57-127.03)	0.66	127.96 (126.59-129.33)	0.74
13-18 years	126.67 (126.06-127.28)	0.07	126.68 (126.06-127.30)	0.08	128.44 (127.13-129.75)	0.05
**Diastolic blood pressure**						
Unexposed	72.90 (72.82-72.99)	-	72.94 (72.81-73.07)	-	73.93 (72.99-74.87)	-
Any loss	72.98 (72.63-73.32)	0.70	72.95 (72.61-73.30)	0.95	73.93 (72.93-74.92)	0.99
Cause of death of the parent						
Unnatural death	72.16 (71.43-72.88)	0.05	72.10 (71.37-72.82)	0.02	73.20 (72.02-74.39)	0.06
Cardiovascular death	73.42 (72.68-74.16)	0.18	73.40 (72.66-74.14)	0.24	74.14 (72.94-75.33)	0.60
Other natural cause	73.13 (72.67-73.58)	0.34	73.12 (72.67-73.58)	0.46	74.14 (73.10-75.18)	0.40
Gender of deceased parent						
Mother	72.39 (71.76-73.02)	0.11	72.43 (71.79-73.06)	0.12	73.52 (72.39-74.66)	0.23
Father	73.22 (72.81-73.63)	0.14	73.17 (72.76-73.58)	0.27	74.09 (73.07-75.11)	0.43
Child’s age at loss						
≤5 years	72.85 (71.99-73.72)	0.91	72.75 (71.87-73.62)	0.66	73.68 (72.40-74.95)	0.57
6-12 years	73.04 (72.46-73.62)	0.66	72.99 (72.40-73.57)	0.89	73.92 (72.81-75.02)	0.95
13-18 years	72.97 (72.49-73.45)	0.80	73.00 (72.50-73.49)	0.84	74.02 (72.97-75.08)	0.74

For peer review only

LS Mean=least square mean; CI=confidence interval.

*Model 1 was unadjusted.

†Model 2 was adjusted for parental socioeconomic status.

‡Model 3 was adjusted for parental socioeconomic status, depression, anxiety, body-mass index, cardiorespiratory fitness, number of friends, frequency of talking with friends confidentially, having a confidant, number of cigarettes smoked per day, and risky drinking behavior.

For peer review only

Supplementary table 3. Associations between death of a parent and blood pressure at conscription, stratified by parental socioeconomic status and childhood adversity (N=48624)

Potential effect modifiers	Systolic blood pressure		Diastolic blood pressure	
	LS Mean (95% CI)*	*P*-value†	LS Mean (95% CI)*	*P*-value†
Parental socioeconomic status				
Non-manual employee	126.75 (124.57-128.92)	-	73.61 (71.84-75.38)	-
Unskilled worker/Skilled worker	129.11 (127.53-130.70)	0.86	73.94 (72.66-75.22)	0.72
Famer/Other	129.61 (125.66-133.56)	0.64	76.09 (72.92-79.26)	0.01
Cumulative childhood adversity				
0	126.61 (122.78-130.44)	-	75.47 (72.38-78.56)	-
1	130.75 (128.58-132.92)	0.84	74.41 (72.66-76.16)	0.16
2	127.95 (125.56-130.35)	0.68	74.08 (72.16-76.00)	<0.01
≥3	127.37 (125.14-129.60)	0.96	73.00 (71.17-74.83)	0.13

LS Mean=least square mean; CI=confidence interval.

For peer review only

*Models were adjusted for parental socioeconomic status, depression, anxiety, body-mass index, cardiorespiratory fitness, number of friends, frequency of talking with friends confidentially, having a confidant, number of cigarettes smoked per day, risky drinking behavior and cumulative childhood experience.

†*P*-value for interaction between death of a parent and the potential effect modifier.

For peer review only

STROBE 2007 (v4) Statement—Checklist of items that should be included in reports of *cohort studies*

Section/Topic	Item #	Recommendation	Reported on page #
**Title and abstract**	1	(*a*) Indicate the study's design with a commonly used term in the title or the abstract	1
		(*b*) Provide in the abstract an informative and balanced summary of what was done and what was found	2
**Introduction**			
Background/rationale	2	Explain the scientific background and rationale for the investigation being reported	4-6
Objectives	3	State specific objectives, including any prespecified hypotheses	6
**Methods**			
Study design	4	Present key elements of study design early in the paper	6
Setting	5	Describe the setting, locations, and relevant dates, including periods of recruitment, exposure, follow-up, and data collection	6
Participants	6	(*a*) Give the eligibility criteria, and the sources and methods of selection of participants. Describe methods of follow-up	6
		(*b*) For matched studies, give matching criteria and number of exposed and unexposed	NA
Variables	7	Clearly define all outcomes, exposures, predictors, potential confounders, and effect modifiers. Give diagnostic criteria, if applicable	7-9
Data sources/ measurement	8*	For each variable of interest, give sources of data and details of methods of assessment (measurement). Describe comparability of assessment methods if there is more than one group	7-9
Bias	9	Describe any efforts to address potential sources of bias	6-9
Study size	10	Explain how the study size was arrived at	6
Quantitative variables	11	Explain how quantitative variables were handled in the analyses. If applicable, describe which groupings were chosen and why	7-9
Statistical methods	12	(*a*) Describe all statistical methods, including those used to control for confounding	9-10
		(*b*) Describe any methods used to examine subgroups and interactions	9-10
		(*c*) Explain how missing data were addressed	10
		(*d*) If applicable, explain how loss to follow-up was addressed	6
		(*e*) Describe any sensitivity analyses	9-10
**Results**			

For peer review only

Participants	13*	(a) Report numbers of individuals at each stage of study—eg numbers potentially eligible, examined for eligibility, confirmed eligible, included in the study, completing follow-up, and analysed	6
		(b) Give reasons for non-participation at each stage	6
		(c) Consider use of a flow diagram	NA
Descriptive data	14*	(a) Give characteristics of study participants (eg demographic, clinical, social) and information on exposures and potential confounders	11, 24-26
		(b) Indicate number of participants with missing data for each variable of interest	24-26
		(c) Summarise follow-up time (eg, average and total amount)	NA
Outcome data	15*	Report numbers of outcome events or summary measures over time	11
Main results	16	(*a*) Give unadjusted estimates and, if applicable, confounder-adjusted estimates and their precision (eg, 95% confidence interval). Make clear which confounders were adjusted for and why they were included	11, 27-36
		(*b*) Report category boundaries when continuous variables were categorized	11, 27-36
		(*c*) If relevant, consider translating estimates of relative risk into absolute risk for a meaningful time period	NA
Other analyses	17	Report other analyses done—eg analyses of subgroups and interactions, and sensitivity analyses	11
**Discussion**			
Key results	18	Summarise key results with reference to study objectives	12
**Limitations**			
Interpretation	20	Give a cautious overall interpretation of results considering objectives, limitations, multiplicity of analyses, results from similar studies, and other relevant evidence	12-16
Generalisability	21	Discuss the generalisability (external validity) of the study results	15
**Other information**			
Funding	22	Give the source of funding and the role of the funders for the present study and, if applicable, for the original study on which the present article is based	16

*Give information separately for cases and controls in case-control studies and, if applicable, for exposed and unexposed groups in cohort and cross-sectional studies.

Note: An Explanation and Elaboration article discusses each checklist item and gives methodological background and published examples of transparent reporting. The STROBE checklist is best used in conjunction with this article (freely available on the Web sites of PLoS Medicine at http://www.plosmedicine.org/, Annals of Internal Medicine at http://www.annals.org/, and Epidemiology at http://www.epidem.com/). Information on the STROBE Initiative is available at www.strobe-statement.org.

For peer review only
